# Human antigen R-regulated mRNA metabolism promotes the cell motility of migrating mouse neurons

**DOI:** 10.1242/dev.183509

**Published:** 2020-03-16

**Authors:** Yi-Fei Zhao, Xiao-Xiao He, Zi-Fei Song, Ye Guo, Yan-Ning Zhang, Hua-Li Yu, Zi-Xuan He, Wen-Cheng Xiong, Weixiang Guo, Xiao-Juan Zhu

**Affiliations:** 1 Key Laboratory of Molecular Epigenetics, Ministry of Education, Institute of Genetics and Cytology, Northeast Normal University, Changchun 130024, China; 2 State Key Laboratory for Molecular and Developmental Biology, Institute of Genetics and Developmental Biology, Chinese Academy of Sciences, Beijing 100101, China; 3 Department of Neurosciences, Case Western Reserve University, School of Medicine, Cleveland, OH 44106, USA; 4 Graduate School, University of Chinese Academy of Sciences, Beijing 100093, China

**Keywords:** HuR, Profilin1, Neocortex development, Neuronal migration, Actin polymerization, mRNA metabolism

## Abstract

Neocortex development during embryonic stages requires the precise control of mRNA metabolism. Human antigen R (HuR) is a well-studied mRNA-binding protein that regulates mRNA metabolism, and it is highly expressed in the neocortex during developmental stages. Deletion of HuR does not impair neural progenitor cell proliferation or differentiation, but it disturbs the laminar structure of the neocortex. We report that HuR is expressed in postmitotic projection neurons during mouse brain development. Specifically, depletion of HuR in these neurons led to a mislocalization of CDP^+^ neurons in deeper layers of the cortex. Time-lapse microscopy showed that HuR was required for the promotion of cell motility in migrating neurons. PCR array identified profilin 1 (*Pfn1*) mRNA as a major binding partner of HuR in neurons. HuR positively mediated the stability of *Pfn1* mRNA and influenced actin polymerization. Overexpression of Pfn1 successfully rescued the migration defects of HuR-deleted neurons. Our data reveal a post-transcriptional mechanism that maintains actin dynamics during neuronal migration.

## Abstract (precis)

**Summary:** Maintaining actin dynamics is crucial for cell motility. Post-transcriptional regulation plays a pivotal role in supporting actin dynamics during neuronal migration.

## INTRODUCTION

A correct laminar structure of the mammalian neocortex is essential to maintain proper brain function. Neuronal migration of cortical excitatory neurons (also known as projection neurons) is the key step in the establishment of the six layers of neocortex during embryonic development (Kriegstein and Noctor, 2004; Sakakibara and Hatanaka, 2015; Sultan et al., 2013). Projection neurons follow an inside-out migration pattern to their final destinations in the cortex. Early born neurons that possess a long leading process attached to the pial surface undergo somal translocation to establish the preplate (Nadarajah et al., 2001). Late-born neurons sequentially use three different migration modes, multipolar migration, glia-guided radial locomotion and terminal somal translocation, to reach the upper layer of the cerebral cortex (Rakic, 1972; Tabata and Nakajima, 2003). Neuronal migration is a highly complex and dynamic process that may require several days to complete in the mouse brain. Numerous factors regulate this process, such as cytoskeletal, cell-adhesion and cell-signaling molecules (Ayala et al., 2007; Ohtaka-Maruyama and Okado, 2015). However, mRNA metabolism, which is a fundamental cellular process that contributes to the generation of all of the aforementioned cellular molecules, is not well-studied in neuronal migration.

The RNA-binding proteins (RBPs) are key to the spatiotemporal orchestration of mRNA fate, and mediate mRNA splicing, transportation, stability and translation (Darnell, 2013; Lebedeva et al., 2011). Human antigen R (also known as ELAVL1) is a ubiquitously distributed member of the Hu family. HuR was estimated to have 26,000 transcriptome-wide targets in different cell lines, and it was implicated in multiple steps of post-transcriptional mRNA processing (Katsanou et al., 2003; Lebedeva et al., 2011; Mukherjee et al., 2011). HuR-mediated mRNA metabolism was implicated in neuron protection (Skliris et al., 2015), neurodegeneration disease (Lu et al., 2009; Vanderweyde et al., 2013), synaptic function (Yokoi et al., 2017), depression (He et al., 2019) and adult neurogenesis (Wang et al., 2019) in the adult mouse brain. In contrast, the role of HuR in the developing mouse brain is not well studied, which may be due to lethality in HuR-deleted embryos. Recent studies showed that conditional knockout of HuR in the telencephalon during embryonic development impaired proper neocortex formation (Kraushar et al., 2014; Popovitchenko et al., 2016). Wang and colleagues also showed that HuR had no impact on the proliferation and differentiation of neural progenitor cells (NPCs) (Wang et al., 2019). Therefore, the physiological role of HuR in developing neurons remains obscure.

We found that HuR expression in postmitotic neurons contributed to the laminar structure of the mouse brain. *In utero* electroporation and time-lapse microscopy techniques were used and discovered a specific role of HuR in the regulation of cell motility of migrating neurons. PCR array screening identified the mRNA of profilin 1 (*Pfn1*) as a major binding partner of HuR. Biochemical studies confirmed that HuR regulated the stability of *Pfn1* mRNA, which influenced the F/G-actin ratio in neurons. Notably, Pfn1 overexpression rescued cell motility defects in HuR-deleted neurons. These data showed a crucial role for HuR in the regulation of neuronal migration during brain development.

## RESULTS

### HuR in post-mitotic projection neurons regulates corticogenesis

**Fig. 1.**
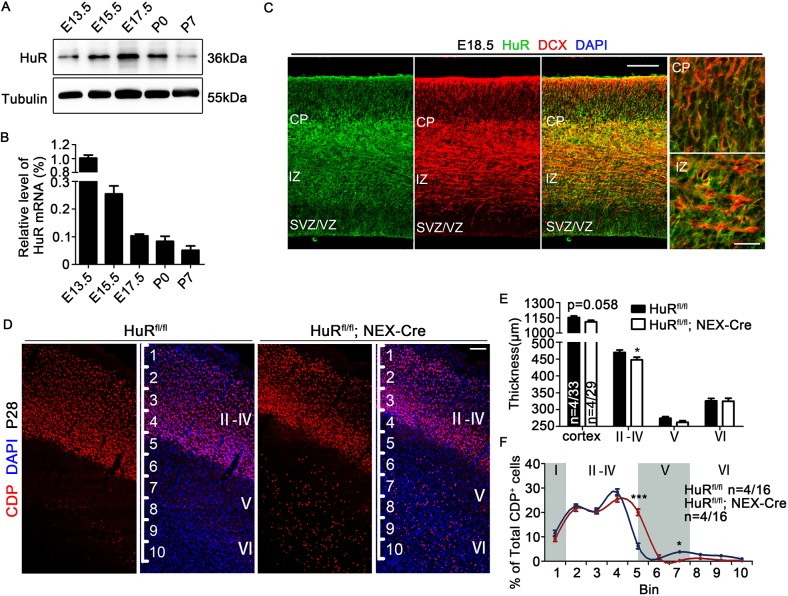
**HuR is expressed in post-mitotic neurons and regulates the laminar structure of the mouse neocortex.** (A) Western blotting analyses of HuR protein expression in different neocortex samples. (B) Real-time PCR analyses of HuR mRNA expression in neocortex samples. Expression levels at E13.5 were set as 1. All experiments were performed three times in triplicate. (C) Immunostaining for HuR and DCX in E16.5 wild-type cortical sections. HuR highly colocalized with DCX in the developing brains. Magnified images of the CP and IZ are shown in the right panels. Scale bars: 100 μm; 20 μm (higher magnification). (D) Immunostaining of CDP in P28 HuR^fl/fl^ and HuR^fl/fl^; NEX-Cre cortical sections. Scale bar: 100 μm. Cerebral cortices were divided into 10 equal bins. Scale bar: 100 μm. (E) Quantification of cortical layer thickness in HuR^fl/fl^ and HuR^fl/fl^; NEX-Cre cerebral cortices. Student's *t*-test was used for statistical analysis. *n* values represent the numbers of brains or brain sections. (F) Distribution of CDP^+^ neurons in the cortex. Two-way ANOVA with Bonferroni's post-hoc analysis was used for statistical analysis. *n* values represent the numbers of brains or brain sections. For all images, nuclei were counterstained with DAPI. The numbers of brains/brain sections quantified in each experiment are indicated on the graphs. Data are mean±s.e.m. ns, not significant, *P*>0.5; *P<0.05; ***P<0.001.

HuR had peak protein levels from E15.5 to E17.5 in the developing mouse neocortex (Fig. 1A), which was consistent with the high mRNA levels of HuR from E13.5 to E15.5 (Fig. 1B), because a two-day delay is often observed for corresponding mRNA and protein levels in the developing cortex (DeBoer et al., 2013; Kwan et al., 2012). Immunostaining results showed ubiquitous distribution of HuR in the developing brain, with a stronger staining signal in the cortical plate (CP) (Fig. 1C). Previous studies have shown that deletion of HuR disturbed lamination of the mouse neocortex (Kraushar et al., 2014; Popovitchenko et al., 2016), which could result from defects in neurogenesis or neuronal migration. Wang and colleagues recently reported that HuR conditional knockout (KO) by EMX1-Cre did not impair neurogenesis during mouse embryonic development (Wang et al., 2019). Therefore, we first examined proliferation and differentiation of neural progenitor cells (NPCs) in HuR^fl/fl^; Nestin-Cre mice. Nestin-Cre mediates recombination in all types of neural progenitor cell and should have a broader effect than EMX1-Cre-mediated gene knockout (Giusti et al., 2014). BrdU labeling was performed at E14.5, and brain slices were analyzed at 2 h and 48 h. The ratios of PAX6^+^ BrdU^+^ and Tbr2^+^ BrdU^+^ cells among the total BrdU^+^ cells were similar between HuR^fl/fl^; Nestin-Cre mice and HuR^fl/fl^ mice 2 h post-BrdU injection (Fig. S1A-E). The ratio of Tbr1^+^ BrdU^+^ cells among total BrdU^+^ cells was also similar between the two mouse brains 48 h post-BrdU injection (Fig. S1F,G). Therefore, HuR most likely functions in post-mitotic neurons to contribute to neocortical lamination. Consistent with this hypothesis, we found that HuR was highly colocalized with DCX, a marker of immature neurons, in the E16.5 developing brain (Fig. 1C). To specifically address the roles of HuR in these neurons, we crossed HuR^fl/fl^ mice with NEX-Cre mice, in which Cre mediates recombination in post-mitotic projection neurons (Goebbels et al., 2006). NEX-Cre-mediated expression was confirmed in brain sections of a reporter mouse (Ai27D; NEX-Cre) (Fig. S2A). Therefore, the HuR^fl/fl^; NEX-Cre mice should lack HuR expression in post-mitotic projection neurons, beginning at E11.5 (Goebbels et al., 2006). A substantial decrease in HuR protein levels was detected in cortical lysates of P0 HuR^fl/fl^; NEX-Cre mice (Fig. S2B), and HuR was absent from CDP^+^ and CTIP2^+^ neurons (Fig. S2C), which are markers of mature cortical neurons. These data confirmed the HuR knockout efficiency and specificity in HuR^fl/fl^; NEX-Cre mice.

Using this mouse model, we studied cortical lamination by co-staining with DAPI and for CDP, CTIP2 or FOXP2 in P28 mouse cortical sections. No differences in total cortical thickness were detected, but a significant decrease in layer II-IV thickness (defined by DAPI staining) was observed in HuR^fl/fl^; NEX-Cre mice (Fig. 1D,E). Consistently, a reduction in CDP^+^ layer thickness was observed in HuR^fl/fl^; NEX-Cre mice (Fig. 1D,F), but not for other markers (Fig. S2D). The distributions of CDP^+^, CTIP2^+^ and FOXP2^+^ cells were quantified in 10 arbitrarily defined layers along the cortex. Statistical analyses showed that substantially more CDP^+^ cells were present in deeper layers (bin 5 and bin 7) of HuR^fl/fl^; NEX-Cre mice compared with the control mice (Fig. 1F). CTIP2 and FOXP2 staining revealed no significant differences between the control and mutant mice (Fig. S2E,F). Therefore, cortical lamination was not properly established when HuR was absent from post-mitotic neurons.

### HuR is essential for neuronal migration of late-born neurons

**Fig. 2.**
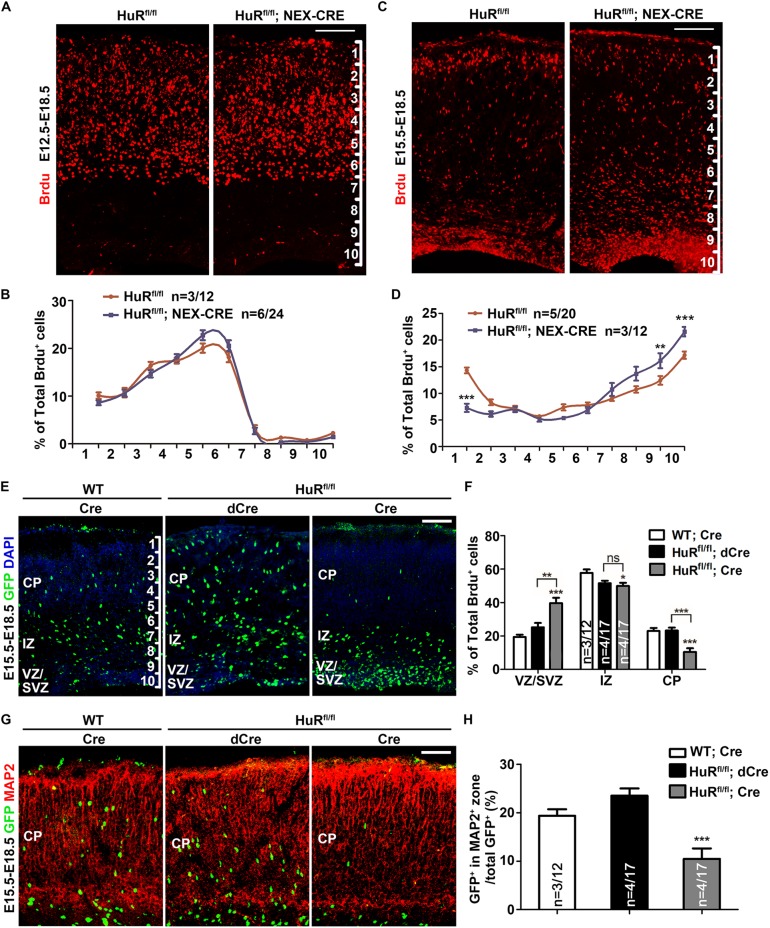
**HuR deficiency impairs the neuronal migration of late-born neurons.** (A) Pregnant mice were administered BrdU at E12.5. Coronal sections of each brain were collected at E18.5 and immunostained for BrdU. Cerebral cortices were divided into 10 equal bins. (B) Distribution of BrdU^+^ cells in the cortex of A. Two-way ANOVA with Bonferroni's post-hoc analysis was used for statistical analysis. *n* values represent the numbers of brains/brain sections. (C) Pregnant mice were administered BrdU at E15.5. Coronal sections of each brain were collected at E18.5 and immunostained for BrdU. Cerebral cortices were divided into 10 equal bins. (D) Distribution of BrdU^+^ cells in the cortex of C. Two-way ANOVA with Bonferroni's post-hoc analysis was used for statistical analysis. *n* values represent the numbers of brains/brain sections. (E) GFP-dCre or GFP-Cre plasmids were electroporated into E15.5 HuR^fl/fl^ or wild-type embryo cortices. Cortical sections were analyzed at E18.5 and immunostained for GFP. Nuclei were counterstained with DAPI. (F) Quantification results of GFP^+^ neuronal distribution across the cerebral cortex. Fewer GFP^+^ neurons were present in the IZ and CP of HuR^fl/fl^; Cre cortical sections. One-way ANOVA with Bonferroni's post-hoc analysis was performed to analyze the data. *n* values represent the numbers of brains/brain sections. (G) dCre or Cre plasmids were electroporated into E15.5 HuR^fl/fl^ or wild-type embryo cortices. Cortical sections were analyzed at E18.5 and immunostained for GFP and MAP2. (H) Quantification of GFP^+^ neuronal distribution in the CP (MAP2 staining zone). One-way ANOVA with Bonferroni's post-hoc analysis was performed to analyze the data. *n* values represent the numbers of brains/brain sections. Data are mean±s.e.m. ns, not significant, *P*>0.5; *P<0.05; **P<0.01; ***P<0.001. Scale bars: 100 μm.

During corticogenesis, post-mitotic neurons follow an inside-out model to migrate to their final destinations (Molnár et al., 2010; Sekine et al., 2011). To further illustrate the role of HuR in neuronal migration, we used a BrdU birthdating assay to compare the laminar position of BrdU^+^ cells in E18.5 mutant mice and their control littermates. BrdU was administered at E12.5 to label early-born neurons (primarily localized in deeper layers) and at E15.5 to label late-born neurons (primarily localized in upper layers). No significant differences were observed in early-born neurons (Fig. 2A,B), but a significant proportion of late-born neurons failed to migrate to the upper layers and stayed in the deeper layers of the cortex (Fig. 2C,D). Therefore, HuR deletion primarily impaired the migration of late-born neurons, which is consistent with the lamination defect of the CDP^+^ layer.

We then used the Cre-flox system to eliminate HuR in late-born neurons via *in utero* electroporation to verify the role of HuR in neuronal migration. RV-CAG-GFP-Cre (GFP-Cre) and RV-CAG-GFP-deltaCre (GFP-dCre) vectors that restricted the GFP localization in nuclei were electroporated into E15.5 HuR^fl/fl^ cortices. A GFP-Cre vector was also electroporated into E15.5 wild-type cortices as a negative control. All mouse brains were analyzed at E18.5 to assess the distribution of GFP^+^ cells. Quantification results showed that substantially more GFP^+^ cells accumulated in the intermediate zone (IZ) and the ventricular/subventricular zone (VZ/SVZ) of HuR knockout (KO) brains than in those of the other two groups (Fig. 2E,F). Correspondingly, significantly fewer GFP^+^ cells presented in the CP (represented by MAP2 staining) of HuR KO brains (Fig. 2G,H).

We constructed shHuR304 and shHuR686 plasmids to knockdown (KD) HuR expression *in vivo*, and a shNC was also constructed as a negative control. Western blotting analyses showed that shHuR686 had better knockdown efficiency than shHuR304 (Fig. S3A). Therefore, shHuR686 was used in the subsequent HuR KD experiments. The shNC or shHuR686 plasmid was electroporated into wild-type cortices at E15.5, and brain slices were analyzed at E18.5 (Fig. S3B). Quantification results showed a significant decrease in GFP^+^ neurons in the CP and a concomitant increase in GFP^+^ neurons in the IZ and VZ/SVZ in the cortices transfected with shHuR686 compared with shNC (Fig. S3B,C). These data indicate that HuR plays an essential role in neuronal migration.

### HuR deficiency impairs the cell motility of migrating neurons

**Fig. 3.**
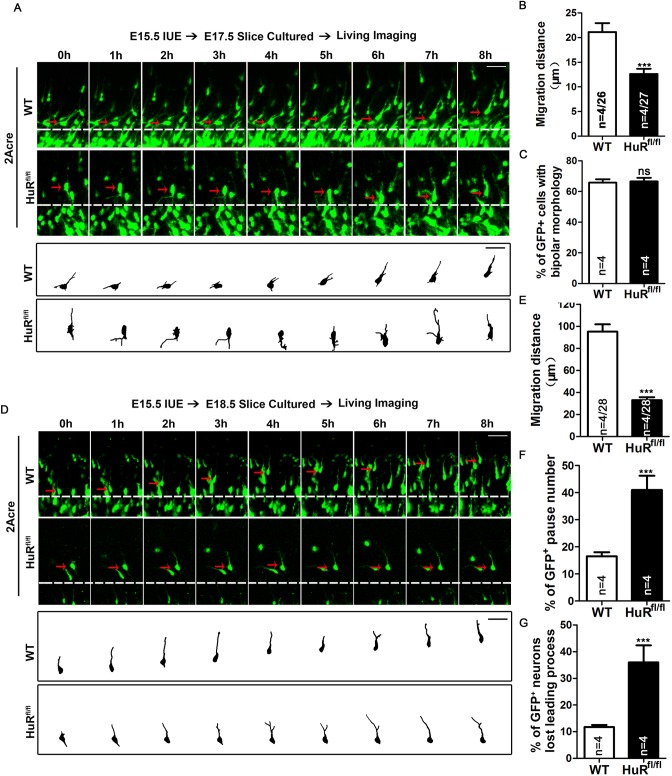
**HuR deficiency impairs the cell motility of migrating neurons.** (A) Brain slices were obtained 2 days after *in utero* electroporation of GFP-2A-Cre plasmids into E15.5 HuR^fl/fl^ or wild-type embryos. An 8 h time-lapse imaging assay was performed for each slice to acquire serial images of migrating neurons in the IZ. Representative neurons are indicated by red arrows; tracings of each neuron are shown in the lower panels. Scale bars: 20 μm. (B) Quantification of migration distance of neurons in A. *n* values represent the numbers of brains/neurons. (C) Percentages of GFP^+^ neurons in A that completed the multipolar-to-bipolar transition during the recording period. *n* values represent the numbers of brain sections. (D) Brain slices were obtained 3 days after *in utero* electroporation of RV-CAG-GFP-2A-Cre plasmids into E15.5 HuR^fl/fl^ or wild-type embryos. An 8 h time-lapse imaging assay was performed for each slice to acquire serial images of migrating neurons in the CP. Representative neurons are indicated by red arrows; tracings of each neuron are shown in the lower panels. Scale bars: 20 μm. (E) Quantification of migration distance of neurons in D. *n* values represent the numbers of brains/neurons. (F) Percentage of GFP^+^ neurons that did not migrate during the entire recording period in D. *n* values represent the numbers of brain sections. (G) Percentage of GFP^+^ neurons that lost a proper leading process during the recording period in D. At least 40 GFP^+^ neurons were counted in each cortical slice. *n* values represent the numbers of brain sections. Data are from four independent experiments. Data are mean±s.e.m. ns, not significant, *P*>0.5; *P<0.05; **P<0.01; ***P<0.001, Student's *t*-test.

Late-born neurons follow a specific sequence of migration modes, including multipolar migration, glia-guided locomotion and terminal somal translocation, to arrive at their correct localizations. Because we found that HuR-deficient neurons accumulated in the IZ and VZ/SVZ, time-lapse microscopy was used to analyze the migration process of neurons. The GFP-2Acre vector, which allows GFP distribution throughout the cytoplasm, was electroporated into E15.5 wild-type and HuR^fl/fl^ cortices to label late-born neurons. Live cortical slices were prepared at E17.5 to visualize the dynamic behaviors of GFP^+^ neurons in the IZ (Fig. 3A and Fig. S4A). The total migration distance of neurons in an 8 h period decreased significantly in HuR-deleted neurons (Fig. 3B). However, the percentages of GFP^+^ neurons that possessed a bipolar morphology were similar between the two electroporated groups (Fig. 3C). Because the multipolar-to-bipolar morphological transition is a crucial step for migrating neurons in the IZ, we further analyzed the morphology of migrating neurons using a shRNA approach. The percentage of bipolar cells in the upper IZ (upIZ) and number of processes per cells in the lower IZ (loIZ) and upIZ were not significantly different between the shHuR686 and shNC groups (Fig. S4B-E).

We next performed an *in utero* electroporation assay at E15.5-E18.5 to investigate whether deletion of HuR affected neuronal migration in the CP. We specifically focused on GFP^+^ neurons that had adopted bipolar morphology in the CP (Fig. 3D and Fig. S4F). The total migration distance of HuR^−^deleted neurons was significantly shorter than that of control neurons (Fig. 3E). We also noticed significantly more static GFP^+^ cells that did not move at all during the recording period in HuR-knockout cortices (Fig. 3F). These cells were not included in the calculation of total migration distance. The HuR-deleted neurons were inclined to the loss and regeneration of the leading process (Fig. 3G). These data suggest that the migration deficits in HuR KO neurons is due to the impairment of cell motility but not defects in morphological transition.

### HuR binds to *profilin1* mRNA to promote Profilin1 expression

**Fig. 4.**
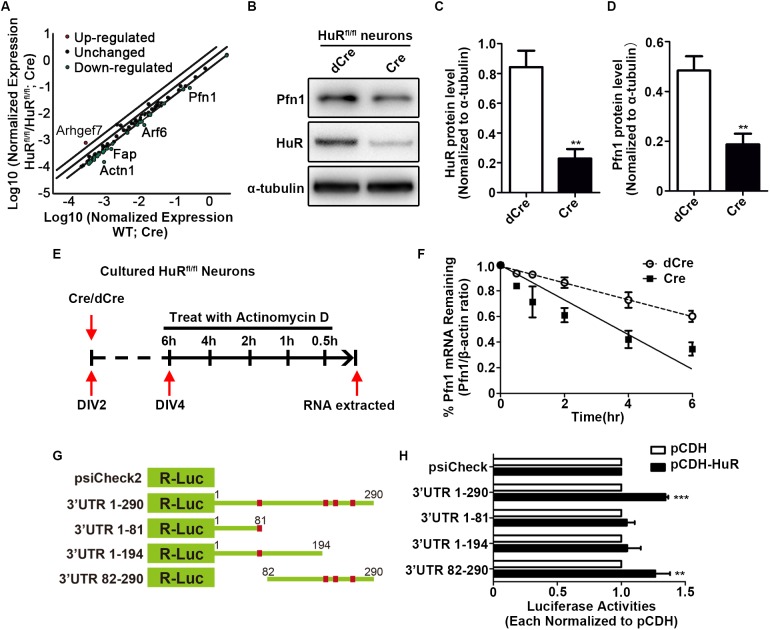
**HuR binds to profilin 1 mRNA to promote profilin 1 expression.** (A) Scatter plot of PCR array results. A two-fold expression change was selected as the threshold. Upregulated genes are highlighted in red; downregulated genes are highlighted in green. (B) Representative western blotting analysis of Pfn1 protein expression in HuR^fl/fl^ neurons infected with GFP-Cre or GFP-dCre lentivirus. (C,D) Quantitative analyses showed that HuR and Pfn1 expression levels were significantly reduced in GFP-Cre-infected HuR^fl/fl^ neurons. Quantification results were from three independent experiments. Student's *t*-test was used for statistical analysis. (E) Schematic of actinomycin D treatment and RNA extraction of Cre and dCre lentivirus-infected neurons. (F) Slope curve of *Pfn1* mRNA metabolism rate. Actinomycin D treatment accelerated *Pfn1* mRNA degradation in neurons. (G) Schematic of all constructs of *Pfn1* mRNA 3′UTRs that were used in dual-luciferase reporter assays. (H) R-Luc activities produced by different constructs were normalized to control F-Luc activities in the same psiCheck2 vectors. Luciferase activities in the pCDH-HuR transfected conditions were compared with the pCDH-transfected conditions. Quantification results are from four independent experiments. Student's *t*-test was used for statistical analysis. Data are mean±s.e.m. **P<0.01; ***P<0.001.

Because HuR is a well-studied mRNA-binding protein, we used a mouse cell motility PCR array to identify target genes that exhibited altered expression levels in HuR KO neurons. Total RNA was extracted from cultured HuR^fl/fl^ and wild-type NPCs infected with GFP-Cre lentivirus. Deletion of HuR in the infected cells was confirmed using immunostaining (Fig. S5A). The results showed that most of the detected genes were downregulated, with a few exceptions of upregulated genes (Fig. 4A and Table S1), which is consistent with the canonical function of HuR as an mRNA stabilizer. To validate this result, we used RNA-IP to confirm the binding of HuR with the target mRNAs (Fig. S5B). Except for the mRNA of *actn3*, most of the tested mRNAs were detected in the HuR-binding complex (Fig. S5C). We also verified the mRNA expression levels of the most downregulated (*Actn1*, *Fap*, *Arf6* and *Pfn1*) and upregulated (*Arhgef7*) genes in cortical samples. The results showed the expression levels of *Arf6* and *Pfn1* were significantly decreased, and the expression level of *Arhgef7* was increased in HuR-deleted cortices, but the expression levels of *Actn1* and *Fap* were not significantly altered (Fig. S5D).

Among the most upregulated and downregulated genes, *Pfn1* triggered our particular interest because it plays well-defined roles in regulating cell motility (Witke, 2004). The phenotypes of *Pfn1* KO mouse neocortices were similar to those of HuR^fl/fl^; NEX-Cre mice (Kullmann et al., 2011, 2012; Popovitchenko et al., 2016). We further verified a positive correlation between HuR and Pfn1 protein levels in HuR KO neurons and HuR KD NLT cells (Fig. 4B-D and Fig. S5E-G). Consistent with our RNA-IP results, the mRNA of *Pfn1* precipitated with HuR protein (Fig. S5H). Therefore, HuR likely binds and stabilizes *Pfn1* mRNA to promote Pfn1 expression. To test this hypothesis, HuR KO and control neurons were incubated with actinomycin D to inhibit mRNA synthesis, and the mRNA stability of *Pfn1* was assessed over a 6 h period (Fig. 4E). The results showed that *Pfn1* mRNA degraded in a time-dependent manner in HuR KO and control neurons (Fig. 4F). However, an almost 50% shorter half-life of *Pfn1* mRNA (T_1/2_=3.78 h in HuR KO cells versus T_1/2_=7.56 h in wild-type cells) was found in HuR KO cells compared with that in control cells (Fig. 4F). These results confirmed that HuR maintained *Pfn1* mRNA stability to ensure proper protein expression in neurons.

The Hu protein family members, including HuR, recognize and bind AU-rich RNA elements (AREs) (Chen et al., 2002). We next investigated whether HuR directly bound with the *Pfn1* mRNA 3′UTR and which part of the sequence was responsible for this binding. ARE sequence analysis was performed, and we cloned the full sequence of the *Pfn1* mRNA 3′UTR and three truncated 3′UTRs, 3′UTR 1-81, 3′UTR 1-194 and 3′UTR 82-290, into a psiCheck2 luciferase reporter vector (Fig. 4G). Each construct was co-transfected with pCDH-HuR into HEK293T cells to assess the activity of luciferase, which was normalized to the same psiCheck2 vector that was co-transfected with pCDH. Significantly elevated luciferase activities were observed for the full *Pfn1* mRNA 3′UTR and for 3′UTR 82-290; 3′UTR 1-81 and 3′UTR 1-194 did not produce this effect (Fig. 4H). Therefore, the latter part of *Pfn1* mRNA 3′UTR was responsible for binding to HuR. Taken together, these data indicated that HuR was a direct stabilizer of *Pfn1* mRNA, which enhanced the protein level of Pfn1 in neurons.

### Profilin 1 rescues migration defects of HuR-deficient neurons

**Fig. 5.**
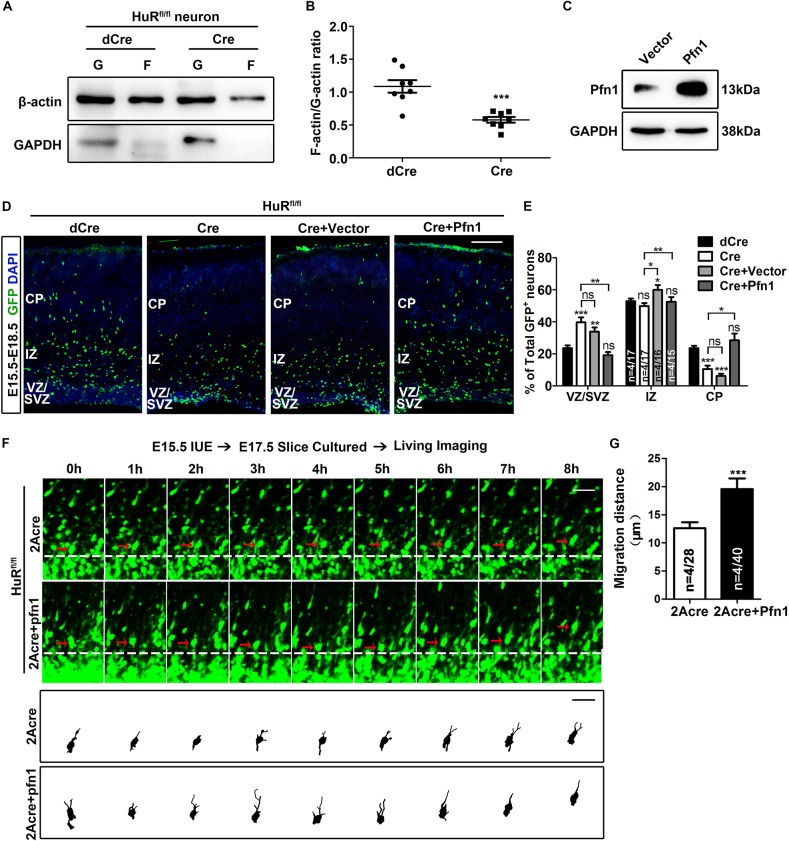
**Profilin 1 rescues the neuronal migration defects of HuR-deleted neurons.** (A) Western blotting analyses of G-actin and F-actin fractions in cultured HuR^fl/fl^ neurons that were infected with dCre and Cre lentivirus. The fraction of F-actin was obviously decreased in HuR-deleted neurons. (B) Quantification of G/F-actin ratio, as determined using densitometry analyses of western blotting results from four independent experiments run in duplicate. Mann–Whitney U-test was used for statistical analysis. (C) NLT cells were transfected with the indicated plasmids. Cell lysates were incubated with an anti-Pfn1 antibody to detect the overexpression of Pfn1. NAPDH was used as a loading control. (D) E15.5 HuR^fl/fl^ embryonic brains were electroporated with the indicated plasmids, and cortical slices were stained with an anti-GFP antibody and DAPI at E18.5. The molar ratio of Cre vector to Pfn1 expression vector is 1:1. Scale bar: 100 μm. (E) Quantification analyses of GFP^+^ neuron distribution in D. One-way ANOVA with Bonferroni's post-hoc analysis was performed to analyze the data. *n* values represent the numbers of brains/brain sections. (F) Brain slices were obtained 2 days after *in utero* electroporation of GFP-2A-Cre or GFP-2A-Cre plus Pfn1 plasmids into E15.5 HuR^fl/fl^ embryos. An 8 h time-lapse imaging assay was performed for each slice. Representative neurons are indicated by red arrows; tracings of each neuron are shown in the lower panels. The molar ratio of 2A-Cre vector to Pfn1 expression vector is 1:1. Scale bars: 20 μm. (G) Quantification of migration distance of neurons in F. Student's *t*-test was used for statistical analysis. *n* values represent the numbers of brains/neurons. Data are mean±s.e.m. ns, not significant, *P*>0.5; *P<0.05; **P<0.01; ***P<0.001.

Pfn1 is a well-known actin-binding protein that regulates F-actin remodeling (Carlsson et al., 1977; Mockrin and Korn, 1980; Witke, 2004), which is crucial in the mediation of cell motility. Therefore, we performed protein fractionation of F-actin and G-actin to test whether the G/F actin ratio was altered in HuR KO neurons. An obvious decrease in F-actin was observed in HuR KO neurons (Fig. 5A,B), which confirmed that HuR was essential for F-actin dynamics in neurons. To validate whether the HuR-mediated actin polymerization defect depended on Pfn1, we constructed a Pfn1 expression vector (Pfn1) and confirmed Pfn1 expression in NLT cells using western blotting (Fig. 5C). The genetic rescue experiment was first performed in NLT cells via co-electroporation of Pfn1 with the shHuR686 plasmid. The results showed that Pfn1 significantly rescued the F-actin deficit in HuR KD cells (Fig. S6A,B). We then co-electroporated the GFP-Cre plasmid with Pfn1 or control vector into HuR^fl/fl^ cortices. The quantification results clearly showed that the distribution of GFP^+^ cells in the cortical slices of the Pfn1 rescue group was similar to that in the control group (Fig. 5D,E). However, when Pfn1 was overexpressed alone in migrating neurons, an accumulation of GFP^+^ cells in the IZ was observed (Fig. S5C,D).

We also performed time-lapse imaging to visualize whether Pfn1 overexpression improved the cell motility defects in HuR KO neurons. GFP-2Acre and Pfn1 expression vectors were co-electroporated into E15.5 HuR^fl/fl^ cortices, and live cortical slices were prepared at E17.5 to analyze the dynamic behaviors of GFP^+^ neurons in the IZ (Fig. 5F). As expected, the total migration distance of GFP^+^ neurons in an 8 h period significantly improved (Fig. 5G), but the total migration distance of these neurons remained less than that of the wild-type control neurons. Considering the broad effect of HuR, we speculated that other downstream mRNAs were also involved in HuR-mediated regulation of neuronal migration. We further tested whether Arf6, which regulates migration speed in the IZ (Hara et al., 2016), rescued the migration defects in HuR-deficient neurons. The results showed that the co-expression of Arf6 did not improve the neuronal migration defects in HuR KO cells (Fig. S6E-G). Collectively, our data provide evidence that Pfn1 is a major target of HuR in the regulation of cell motility of migrating neurons.

## DISCUSSION

The present study demonstrates that HuR is crucial for neuronal migration to establish correct cortical lamination. Furthermore, we provide evidence that HuR mediates cell motility of migrating neurons via regulation of *Pfn1* mRNA stability. These data describe a previously undefined function of HuR during brain development.

Unlike other Hu protein family members (HuB, HuC and HuD) that are specifically expressed in the neural system, HuR is expressed in a wide range of cell types and mediates numerous biological events (Hinman and Lou, 2008). Global deletion of HuR caused the death of mouse embryos beyond mid-gestation (Katsanou et al., 2003), which suggests that HuR played an essential role during embryo development. However, when HuR was deleted in post-mitotic neurons (mediated by NEX-Cre) or in neuroepithelial cells (mediated by FOXG1-Cre), similar lamination defects were observed. Therefore, HuR primarily functions in post-mitotic neurons during neocortex development.

The proper migration of post-mitotic neurons is crucial to establish correct cortical lamination (Rakic, 1972). We found that HuR deletion impaired proper cortex formation, which was shown by the reduced thickness of layers II-IV. In contrast, the deeper layers of the cortex were maintained. Consistent with these data, the BrdU birth-dating assays showed that the migration of late-born neurons (E15.5), but not early-born neurons (E12.5), was impaired significantly. Two reasons may account for the differential roles HuR plays in early-born and late-born neurons. The protein levels of HuR were most prominent in E15.5 and E17.5 brains, which corresponds to the development of late-born neurons. Therefore, HuR should have a more sophisticated impact in these neurons. Notably, we found that the cortical lamination defects in HuR^fl/fl^; Nex-Cre brains primarily resulted from the impaired cell motility of HuR KO neurons. During brain development, late-born neurons travel a much longer distance to arrive at their proper positions in the cortex. Therefore, the requirement for HuR-mediated cell motility was more imperative in these cells.

HuR-mediated cell motility has been well-documented in different cells due to different mechanisms (Chang et al., 2017; Cooper, 1991; Doxakis, 2014; Joseph et al., 2014). We verified *Pfn1* mRNA as a binding partner of HuR in neurons. Pfn family members are pivotal in the promotion of actin dynamics at the plasma membrane to drive actin-linking processes (Carlsson et al., 1977; Witke, 2004). Pfn1 has been shown to regulate cell movement and morphogenesis (Carlsson et al., 1977; Tilney et al., 1983). Pfn1 regulates the size of the neocortex in the nervous system, but no detailed examination of cortical lamination has been performed (Kullmann et al., 2011). We show that Pfn1 plays a major role in HuR-regulated cell motility because the overexpression of Pfn1 successfully rescues the migration defects in HuR KO neurons, and the overexpression of Arf6 does not produce this beneficial effect on neuronal migration. Moreover, Pfn1 overexpression alone has a deleterious effect on neuronal migration. Therefore, the precise control of *Pfn1* mRNA during neuronal migration is crucial. Consistent with our data, HuR deficiency causes defects in migration rate and reduces the number of radial lamellipodia in Schwann cells, which result from Pfn1 downregulation (Iruarrizaga-Lejarreta et al., 2012). In addition, HuR and Pfn1 are both involved in familial amyotrophic lateral sclerosis (Lu et al., 2009; Wu et al., 2012; Brettle et al., 2015; Matsye et al., 2017).

However, HuR, as a RNA-binding protein, may target hundreds of genes for post-transcriptional regulation at any given time. Other genes associated with cell motility may also be involved in the control of neuronal migration. In addition to *Pfn1* and *Arf6*, many other genes that appeared in the PCR array regulate neuronal migration. For example, Rac1 and Cdc42 are small GTPases that are required for precise control during neuronal migration because constitutively active and dominant-negative forms of Rac1 and Cdc42 significantly inhibit radial migration (Konno et al., 2005). The functions of these two molecules are primarily related to malformation of the leading process (Kawauchi et al., 2006; Kholmanskikh et al., 2006; Yang et al., 2012). Rnd3 is an atypical Rho GTPase that antagonizes RhoA activity during neuronal migration. Rnd3-silenced neurons exhibit enlarged leading processes and an excess of thin processes that extend from the cell body and the leading process (Pacary et al., 2011). These GTPases regulate the leading process of migrating neurons, but these phenotypic defects were not present in HuR-deficient neurons, which indicates that the functions of these GTPases do not overlap with HuR or Pfn1. Similar to Pfn1, several actin-binding proteins also play roles in neuronal migration. Myo10 is a nontraditional myosin family member that is involved in establishing proper migration orientation and the multipolar-to-bipolar morphology transition (Ju et al., 2014). The Arp2/3 complex is an actin nucleator that produces branched actin networks, and is essential for the maintenance of radial glial cell (RGC) polarity and organization (Wang et al., 2016). Caveolin 1 regulates clathrin-independent endocytosis and elongation of the leading process (Shikanai et al., 2018). Cofilin disassembles actin filaments, and is negatively regulated by the CDK5-p27 pathway. An appropriate balance of cofilin phosphorylation is required for proper cortical neuronal migration (Kawauchi et al., 2006). However, these phenotypic defects were not similar to our observations in HuR-deficient neurons and brains. Notably, the expression of most of the aforementioned genes was not significantly changed in our PCR array. Therefore, HuR might not regulate the mRNA stability of these genes, and these genes might not play significant roles in HuR-mediated neuronal migration. In conclusion, our study reveals that HuR-mediated cell motility is essential for the development of the neocortex. These data extend the knowledge of the contribution of mRNA metabolism to brain development.

## MATERIALS AND METHODS

### Animals and cell lines

All mice used in this study were handled in accordance with the guidelines of the Institutional Animal Care and Use Committees of the Northeast Normal University, China. Females were maintained on a 12 h light/dark cycle and were bred overnight with males. Noon on the day after breeding was considered E0.5. Wild-type mice were used in primary neuron cultures and *in utero* electroporation was carried out in the C57BL/6 background. HuR^fl/fl^ mice (stock number 021431) and Ai27D (stock number 012567) were initially obtained from The Jackson Laboratory and were maintained in the C57BL/6 background. The NEX-Cre mice (Goebbels et al., 2006) and Nestin-Cre (Giusti et al., 2014) mice used in this study were kind gifts from Dr Zilong Qiu (Institute of Neuroscience, CAS, China). Genotyping of all mice was performed by PCR using primers listed in Table S2.

HEK293T cells were kindly provided by the Stem Cell Bank, Chinese Academy of Sciences. NLT cells were from the stock of our lab, which were originally provided by Dr Wencheng Xiong (Case Western Reserve University, Cleveland, OH, USA). Both cells were cultured in Dulbecco's modified Eagle's medium supplemented with fetal bovine serum (FBS).

### Plasmids

The RV-CAG-GFP-Cre, RV-CAG-GFP-deltaCre, RV-CAG-GFP-2ACre, pCDH-CMV-GFP, pCDH-CMV-GFP-HuR, Lenti-GFP-Cre, Lenti-GFP-delta-Cre, pAX, pMD and psiCheck2 plasmids are stocks from our lab. The shHuR686 was constructed from a pGeneClip hMGFP backbone using the following sequence: 5′-GTGGGATTTCTGGTGTCAATGTC-3′. The shHuR304 was constructed from a pGeneClip hMGFP backbone using the following sequence: 5′-GTCATCAAAGATGCCAACTTAT-3′. The shNC was constructed from the same backbone plasmid with a randomized sequence that has no targets in the mice genome. The pCAGGS-Arf6-flag plasmid was a kind gift from Dr Sakagami (Kitasato University, School of Medicine, Japan). The pCMV-SPORT6-profilin1 plasmid was purchased from RIKEN DNA Bank Human Resource (HGX005814). The truncated *Pfn1* mRNA 3′UTR was constructed in psiCheck2. XhoI and NotI restriction sites are used for digestion and ligation. Sequences of cloning primers are listed in Table S2.

### Antibodies

Primary antibodies included mouse anti-β III Tubulin (1:1000, ab7751, Abcam), anti-β-actin (1:5000, A5316, sigma), rabbit anti-green fluorescent protein (GFP) (1:1000, ab6556, Abcam), mouse anti-MAP2 (1:1000, M1406, Sigma-Aldrich), mouse anti-HuR (1:500, sc5261, Santa Cruz), rabbit anti-CDP (1:500, SC-13024, Santa Cruz), rat anti-CTIP2 (1:500, ab18465, Abcam), rabbit anti-FOXP2 (1:500, ab16046, Abcam), rabbit anti-GABA (1:500, A2052, Sigma), rabbit anti-Ki67 (1:500, RM-9106-S, Thermo Fisher Scientific), rabbit anti-Pax6 (1:200, PRB-278P, Covance Research), rabbit anti-Tbr2 (1:500, ab23345, Abcam), mouse anti-GAPDH (1:5000, HC301-01, TransGene), rat anti-BrdU (1:1000, ab6326, Abcam), rabbit anti-profilin1 (1:1000, ab50667, Abcam). Nuclei were stained with 4′,6-diamidino-2-phenylindole (DAPI) (Roche).

### Western blotting

Fresh brain tissues, transfected HEK293T cells or infected cultured neurons were rinsed with phosphate-buffered saline (PBS) 48 h after transfection and then homogenized in lysis buffer [RIPA: 20 mM Tris-HCl (pH 7.4), 100 mM NaCl, 1% NP-40, 1 mM EDTA, 5 μg/ml aprotinin and 5 μg/ml leupeptin]. Following the addition of SDS sample buffer, samples were boiled for 5 min, equal amounts of protein were subjected to SDS-polyacrylamide-gel electrophoresis (SDS-PAGE) and transferred to polyvinylidene difluoride membranes (Immobilon P; Millipore). Membranes were blocked using 5% bovine serum albumin prepared in TBS-T [50 mM Tris-HCl (pH 7.4), 150 mM NaCl, 0.1% Tween-20] for 1 h at room temperature. Membranes were incubated with corresponding primary antibodies overnight at 4°C then washed three times with TBS-T and incubated with secondary antibody conjugated to horseradish peroxidase for 1.5 h at room temperature. The blotted membranes were developed using Amersham ECLTM Prime Western Blotting Detection Reagent (GE Healthcare).

### Standard PCR, real-time PCR and PCR array

Standard PCR was performed as described previously (Wang et al., 2015). Briefly, each reaction contained 20-40 ng of cDNA (except the cDNA for the IP, for which 5% of the cDNA was used for each gene examined), 1 U GoTaq DNA polymerase (Promega, M3005), and 300 nM of forward and reverse primers (shown below) in a final reaction volume of 20 μl. Data analysis was carried out using Tanon 1600 built in software.

Real-time PCR and data analyses were performed according to the manufacturer's protocol for the SYBR Green PCR supermix (Bio-Rad, 172-5124) using a Real-Time PCR System (Themo PIKOREAL 96). For data analyses, the relative expression level of each gene was normalized to internal controls of the same sample. The relative expression levels of genes between different samples were then calculated, while the expression level of each gene in control was set as 1.

For the PCR array, 10 ng cDNA was added into each well of a mouse cell motility PCR array kit (Qiagen, PAMM-128Z). Each sample was applied to one array and independent duplicates were analyzed for wild-type and HuR KO samples. The sequences of qPCR primers used in this study are listed in Table S2.

### Immunohistochemistry

Animals were transcardially perfused with 4% paraformaldehyde in PBS under anesthesia (0.7% pentobarbital sodium), and brains were fixed in 4% paraformaldehyde in 0.1 M phosphate buffer (pH 7.4) overnight and then placed in 35% sucrose in phosphate-buffered saline (pH 7.4) overnight. Dehydrated samples were embedded in OCT compound (Tissue-Tek) and then cryosectioned using a Leica cryostat CM1950. Immunostaining was performed using standard protocols.

Briefly, for staining, floating and sliced brain sections were first washed in PBS to remove cryoprotectant and then blocked in the PBS buffer containing 2% BSA and 0.2% Triton-X 100, followed by incubation with primary antibodies diluted in PBS overnight in 4°C. After washing three times with PBST, secondary antibodies were incubated for 1.5 h at room temperature. All sections were counterstained with DAPI.

### *In utero* electroporation

*In utero* electroporation was performed as previously described (Tabata and Nakajima, 2001). Briefly, pregnant mice were anesthetized, and their uterine horns were exposed with a midline laparotomy incision. Embryos were removed carefully and placed on humidified gauze pads. Plasmid DNA plus 0.01% Fast Green (Fluka) was injected into lateral ventricles of embryonic brains with a glass micropipette. A volume of 1 μl of shRNA plasmids (2 μg/μl) or expression constructs (2 μg/μl) was injected with plasmid that expressed chicken β-actin promoter-enhanced green fluorescent protein (EGFP). For rescue experiments, expression constructs were co-injected with shRNA or Cre, and CAG-EGFP plasmids. The best rescue concentration was normally screened at molar ratios of 2:1 and 1:1 (Cre vector to expression vector). The best rescue experiment is shown in the results and the molar ratio used for rescue is presented in the figure and figure legends.

For electroporation, 5×50 ms, 37 V square pulses separated by 950 ms intervals were delivered with forceps-type electrodes connected to an ECM 830 electroporator (BTX Harvard Apparatus). The uterus was then replaced in the abdominal cavity, and the abdomen wall and skin were sutured using a surgical needle and thread. The entire procedure was completed within 40 min. The pregnant mice were warmed in an incubator until they regained consciousness, and embryos were allowed to develop *in utero* according to the experimental design.

### Time-lapse imaging

*In utero* electroporation was performed as described above at E15.5. Two days or three days after electroporation, embryonic brains were dissected in cold artificial cerebrospinal fluid. Brain slices (300 μm) were sectioned using a Leica Vibratome VT1000. To visualize neuronal migration, slices were transferred onto Millicell inserts (Millipore) in neurobasal medium (Invitrogen) containing 2% B-27 supplement, 2 mM L-glutamine and penicillin/streptomycin (50 μg/ml). The glass-bottomed dish was then fitted into a temperature-controlled chamber on the microscope stage for 8 h at 37°C under a 5% CO_2_/air atmosphere. Live-cell imaging was recorded every 20 min using an Olympus FV1000 laser scanning confocal microscope.

### Isolation and analyses of NPCs

Embryo-derived NPCs were isolated from E15.5 HuR^fl/fl^ mice and wild-type littermates, and cultured as described previously (Guo et al., 2012). Briefly, mouse cortex was dissected and placed in Hank's balanced salt solution (HBSS) on ice. Tissue was spun down and digested using PBS containing 0.125% (w/v) trypsin (Sigma). After dissociation with a fire-polished glass pipette, cells were filtered through a 70 µm cell strainer (BD Falcon, 252350) and washed with HBSS. The single-cell suspension from each sample was collected and cultured in proliferating medium in a 5% CO_2_ incubator at 37°C. Proliferating medium contained neurobasal medium with B27 serum-free (Invitrogen, 17504-044) and was supplemented 20 ng/ml basic fibroblast growth factor (FGF-2; PeproTech, K1606), 20 ng/ml epidermal growth factor (EGF, PeproTech, A2306), 1% Antibiotic-Antimycotic and 2 mM L-glutamine. Half of the medium was replaced every 2 days.

### Primary cortical neuronal cultures

Primary cortical neurons were cultured as described previously (Zhu et al., 2007). Briefly, embryonic (E16.5) cerebral cortices removed from pregnant mice were chopped into small pieces after the meninges were completely removed. After incubation in PBS containing 0.125% (w/v) trypsin (Sigma) for 20 min at 37°C, digested tissues were mechanically dispersed by repeated passaging through a Pasteur pipette in PBS containing 0.05% (w/v) DNase (Sigma). Dissociated cells were suspended in neurobasal medium supplemented with B-27 (Life Technologies) and penicillin/streptomycin (100 U/ml), and were plated onto poly-D-lysine-coated dishes (Corning) to incubate at 37°C in a 5% CO_2_ atmosphere.

### Packaging of lentivirus

Briefly, lenti-viral DNA was co-transfected with packaging plasmids pMD and pAX into HEK293T cells using the calcium phosphate method. The viral transfection vector DNA and packaging plasmid DNA were transfected into 5×15 cm dishes of cultured HEK293T cells using the calcium phosphate method. The medium containing lentivirus was collected at 36 and 60 h post-transfection, pooled, filtered through a 0.2-µm filter and concentrated using an ultracentrifuge at 20,000 rpm for 2 h at 4°C using a SW27 rotor (Beckman). The virus was washed once and then suspended in 100 µl PBS.

### RNA immunoprecipitation and *Pfn1* RNA binding assay

RNA-IP was performed as described previously (Wang et al., 2015). Briefly, wild-type and HuR KO NPCs were harvested and homogenized in 1 ml of ice-cold lysis buffer [10 mM HEPES (pH 7.4), 200 mM NaCl, 30 mM EDTA and 0.5% Triton X-100] with 2×complete protease inhibitors (Roche, 11873580001). Nuclei and debris were pelleted at 3000 ***g*** for 10 min; the supernatant was collected and raised to 300 mM NaCl, and clarified at 14,000 ***g*** for 30 min. The resulting supernatant was pre-cleared for 1 h with 100 μl recombinant protein G agarose (Invitrogen) (washed with lysis buffer first). An aliquot of pre-cleared input was saved for RNA extraction (200 μl) and protein analysis (100 μl). A monoclonal antibody against HuR was incubated with recombinant protein A dynabeads at 4°C for 2 h and washed three times with lysis buffer. RNase inhibitors (Roche) were added to the remaining lysates. The pre-cleared lysates were immunoprecipitated with antibody-coated recombinant protein G agarose at 4°C for 2 h. After a third wash with the lysis buffer, 10% of immunoprecipitate was saved for protein analysis. The remaining was washed one more time and was re-suspended by Trizol (Invitrogen, 15596018) for RNA isolation.

RNA-binding assay was performed as previously described (Guo et al., 2018). Briefly, the sequence of *Pfn1* was amplified from pCMV-SPORT6-profilin1 using the primers in Table S2. The PCR fragment was then ligated into pEASY-blunt3 vector (Transgene, CB301) in both directions (confirmed by DNA sequencing). Both resultant vectors were then linearized using SacI restriction enzyme to create antisense or sense templates for *in vitro* transcription. Linearized vectors were *in vitro* transcribed using AmpliScritbeTM T7-FlashTM biotin-RNA transcription Kit (ASB71110, Epicentre Biotechnologies). To determine whether synthetic biotinylated *Pfn1* RNA fragments could bind endogenous HuR, protein lysate was prepared by homogenizing mouse hippocampi using RIPA buffer [50 mM Tris-HCl (pH 8), 150 mM NaCl, 1% NP-40, 0.5% sodium deoxycholate, 0.1% SDS]. Purified biotinylated *Pfn1* transcripts (30 μg) were then incubated with 500 μg of total protein in the binding buffer [10 mM HEPES (pH 7.4), 3 mM MgCl_2_, 5% glycerol and 1 mM DTT] for 30 min at room temperature to allow the binding of biotinylated RNA and their binding proteins. Yeast transfer RNA (50 ng/ml) and heparin (5 mg/ml) were then added to the mixture to block non-specific binding and the binding reaction was allowed to continue for 10 more minutes. Dynabeads M-280 streptavidin (112-05D, Invitrogen) was then added to the mixture, and incubated with constant mixing overnight at 4°C. To collect the protein bound by biotinylated *Pfn1* RNA fragments, the beads were washed three times with 1×binding buffer and resuspended in 1×binding buffer with SDS gel loading dye to dissolve protein that bound to biotinylated *Pfn1* RNA fragments. The proteins in the pulldown material were identified by western blotting using mouse anti-HuR antibody.

### Actinomycin D treatment and mRNA stability assay

Culture HuR^fl/fl^ cortical neurons were first infected with Lentivirus-Cre to knockout HuR, then treated with 10 μg/ml of actinomycin D (Sigma-Aldrich, A1410) to inhibit gene transcription as described previously (Guo et al., 2011). Treated neurons were collected at designated time points for RNA isolation and real-time PCR analysis. mRNA levels of profiling 1 were normalized to *Gapdh*. RNA decay kinetics and half-life were analyzed using a published method (Bolognani et al., 2010; Perrone-Bizzozero et al., 1993). Briefly, for single rate decays, we used the exponential function Mt=M0e-ƛt [Mt, amount of mRNA at time t; M0, amount of mRNA time t=0; ƛ=(ln2)/T_1/2_ (T_1/2_ is the half-life of the mRNA)].

### Luciferase reporter assay

Transfection of HEK293T was carried out using PEI according to the manufacturer's protocol. Briefly, 1×10^6^/ml HEK293T were plated into 12-well plates for 24 h. For transfection reagents setup, a total of 1μg DNA was added into 50 µl opti-DMEM medium (Invitrogen, 11058-021) followed by the addition of 3 µg PEI transfection reagent. The solution was mixed by pipetting three to five times, incubated for 20 min and then added into each well. 48 h later, luciferase activity was detected using the Dual-Luciferase Reporter 1000 System (Promega, E1980) based on the manufacturer's protocol. Briefly, collected cells were lysed in 100 μl of 1× passive lysis buffer at room temperature for 15 min. 20 μl of the lysate was then added to 100 μl of Luciferase Assay Buffer II and mixed briefly. Firefly luciferase (F-luc) activity was immediately read using a SpectraMax M2E plate reader (Molecular Devices Corp). Next, 100 μl of Stop & Glo Buffer with Stop & Glo substrate was added and mixed briefly. Renilla luciferase (R-luc) activity was immediately read. R-luc activity was normalized to F-luc activity to eliminate the variation of transfection efficiencies.

### Calculation of the G/F-actin ratio

The F/G-actin ratio was determined by western blotting, as previously described (Huang et al., 2013). Briefly, the two forms of actin differ in that F-actin is insoluble, while G-actin is soluble. Primary cultured neurons that infected with lenti-virus or NLT cells that electroporated with indicated plasmids were homogenized in cold lysis buffer [10 mM K_2_HPO_4_, 100 mM NaF, 50 mM KCl, 2 mM MgCl_2_, 1 mM EGTA, 0.2 mM DTT, 0.5% Triton X-100, 1 mM sucrose (pH 7.0)] and centrifuged at 15,000 ***g*** for 30 min. The actin components in the supernatant represented the fraction of G-actin. The insoluble F-actin in the pellet was re-suspended in lysis buffer plus an equal volume of buffer 2 [1.5 mM guanidine hydrochloride, 1 mM sodium acetate, 1 mM CaCl_2_, 1 mM ATP, and 20 mM Tris-HCl (pH 7.5)] and incubated on ice for 1 h to convert F-actin into soluble G-actin, with gentle mixing every 15 min. The samples were centrifuged at 15,000 ***g*** for 30 min. The actin components in this supernatant represented F-actin. Samples from the supernatant (G-actin) and pellet (F-actin) fractions were proportionally loaded and analyzed by western blotting using an anti-actin antibody.

### Microscopy imaging and statistical analysis

For the *in utero* electroporation assay, the BrdU birthdating assay and the neuronal marker staining assay, the brain sections of the primary somatosensory cortex were observed using Olympus FSX100 or Olympus FV1000 microscopes, and analyzed using FV10-ASW 1.7 software (Olympus). Only the brightness, contrast and color balance were optimized after acquisition. For the *in utero* electroporation assay, cell counting was carried out in a 500 μm wide cortical area; at least 100 GFP^+^ cells were counted in each section. For the BrdU birth-dating assay and neuronal marker staining assay, cell counting was carried out in a 200 μm wide cortical area; at least 150 stained cells were counted in each section. Cell counting was performed in blinded fashion.

Cortical sub-regions were identified on the basis of cell density by using DAPI staining. The numbers of multipolar and bipolar cells, length of leading process and migration distance were counted using ImageJ software. Multipolar cells were defined as cells with more than three processes. For migration distance calculation, a migration tracing line was generated by connecting the center points of a given migrating neuron that presented in serial time-lapse images (60 min intervals). The total length of the migration tracing line was calculated using ImageJ software.

Statistical analysis was performed using Student's *t-*test and Mann–Whitney *U*-test to compare the means of two groups. One-way analysis of variance (ANOVA) with Bonferroni's post-hoc test was used to compare the means of multiple groups. Two-way ANOVA with Bonferroni's post-hoc analysis was used when two variables were included in one statistical analysis (GraphPad Prism and SPSS). Data are presented as the mean±s.e.m. *P<0.05, **P<0.01 and ***P<0.001.

## Supplementary Material

Supplementary information

Reviewer comments
