# Supplementary material for: Human antigen R-regulated mRNA metabolism promotes the cell motility of migrating mouse neurons
Source: Development. 2020 Mar 16;147(6):dev183509. doi: 10.1242/dev.183509 (PMC7097226; doi:10.1242/dev.183509)
Supplement: Supplementary information [file develop-147-183509-s1.pdf]

Supplementary Materials

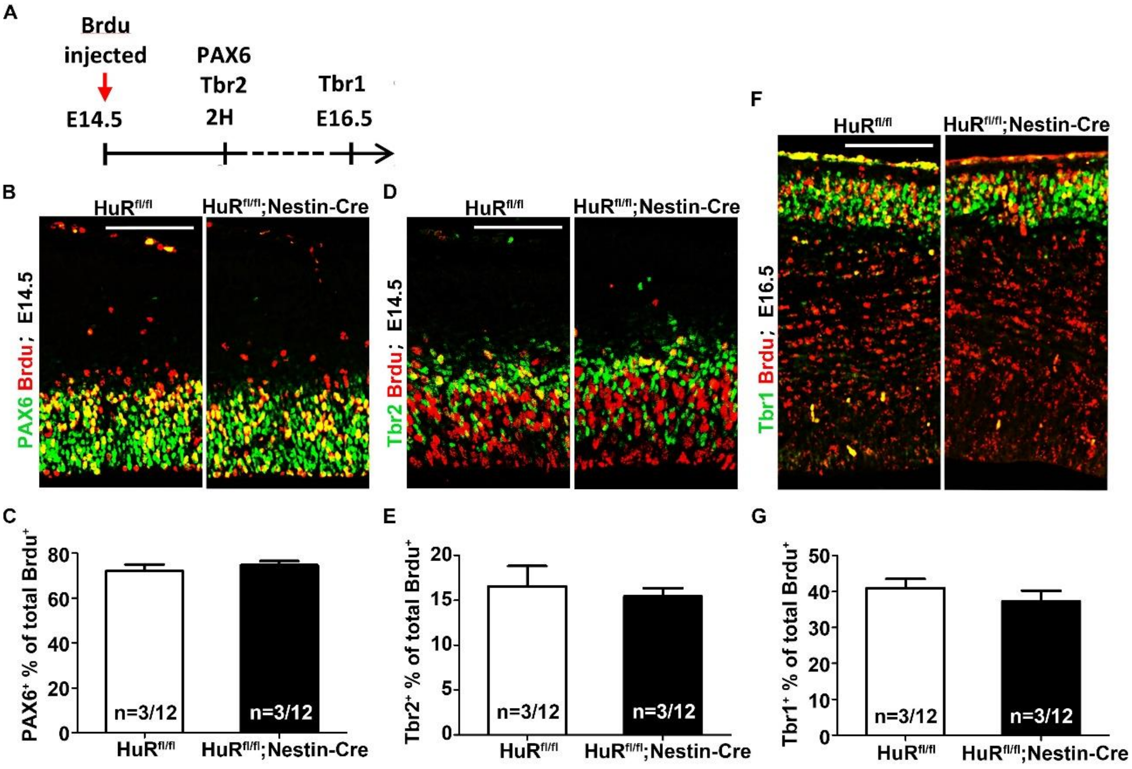

Fig. S1. HuR deletion did not affect neural progenitor cell proliferation or differentiation. (A) Schematic of BrdU labeling assay. (B) BrdU was administrated at E14.5 $HuR^{fl/fl}$; Nestin-Cre mice and $HuR^{fl/fl}$ mice. Brain sections were stained with anti-PAX6 and anti-BrdU antibodies in 2 h post BrdU injection. (C) The ratio of $PAX6^+$ $BrdU^+$ among the total $BrdU^+$ cells was similar between two mice groups. Student's t-test was used for statistical analysis. The n values represent the numbers of brains/brain sections. (D) BrdU was administrated at E14.5 $HuR^{fl/fl}$; Nestin-Cre mice and $HuR^{fl/fl}$ mice. Brain sections were stained with anti-Tbr2 and anti-BrdU antibodies in 2 h post BrdU injection. (E) The ratio of $Tbr2^+$ $BrdU^+$ among the total $BrdU^+$ cells was similar between two mice groups. Student's t-test was used for statistical analysis. The n values represent the numbers of brains/brain sections. (F) BrdU was administrated at E14.5 $HuR^{fl/fl}$; Nestin-Cre mice and $HuR^{fl/fl}$ mice. Brain sections were stained with anti-Tbr1 and anti-BrdU antibodies in 2 h post BrdU injection. (G) The ratio of $Tbr1^+$ $BrdU^+$

Development • Supplementary information

among the total BrdU+ cells was similar between two mice groups. Student's t-test was used for statistical analysis. The n values represent the numbers of brains/brain sections. The data are shown as means ± SEM. n.s.: P > 0.5; *: P < 0.05; **: P < 0.01; ***: P < 0.001.

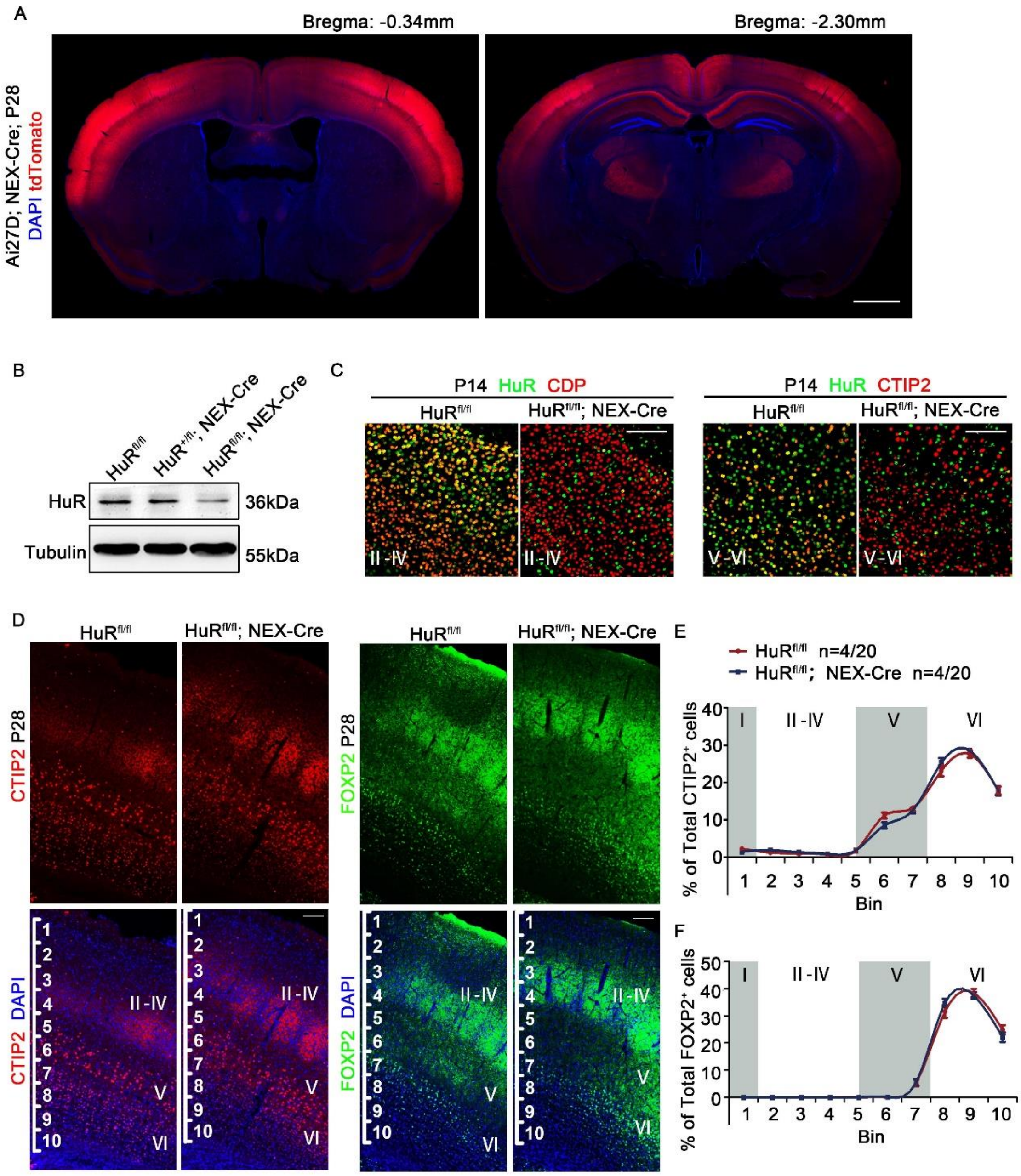

Fig. S2. Deletion of HuR in post-mitotic neurons affects cortical lamination.

(A) Representative images of P28 Ai27D; NEX-Cre cortical sections. The tdTomato signal was present in the cortex and hippocampus. (B) Western blotting analyses of HuR protein expression in P0 HuR$^{fl/fl}$, HuR$^{+/fl}$; NEX-Cre; and HuR$^{fl/fl}$; NEX-Cre

Development • Supplementary information

cortical lysates. (C) Immunostaining of CDP, CTIP2 and HuR in P14 HuR$^{fl/fl}$ and HuR$^{fl/fl}$; NEX-Cre cortical sections. HuR was absent from CDP^{+} and CTIP2^{+} cells. Scale bar=100 μm (D) Immunostaining of CTIP2 and FOXP2 in P28 HuR$^{fl/fl}$ and HuR$^{fl/fl}$; NEX-Cre cortical sections. Scale bar=100 μm. (E) Distribution of CTIP2^{+} neurons in the cortex. Cerebral cortices were divided into 10 equal bins. Two-way ANOVA with *Bonferroni* post-hoc analysis was used for statistical analysis. The n values represent the numbers of brains/brain sections. (F) Distribution of FOXP2^{+} neurons in the cortex. Cerebral cortices were divided into 10 equal bins. Two-way ANOVA with *Bonferroni* post-hoc analysis was used for statistical analysis. The n values represent the numbers of brains/brain sections. For all images in this figure, nuclei were counterstained with DAPI. The data are shown as means ± SEM. n.s.: $P > 0.5$; *: $P < 0.05$; **: $P < 0.01$; ***: $P < 0.001$.

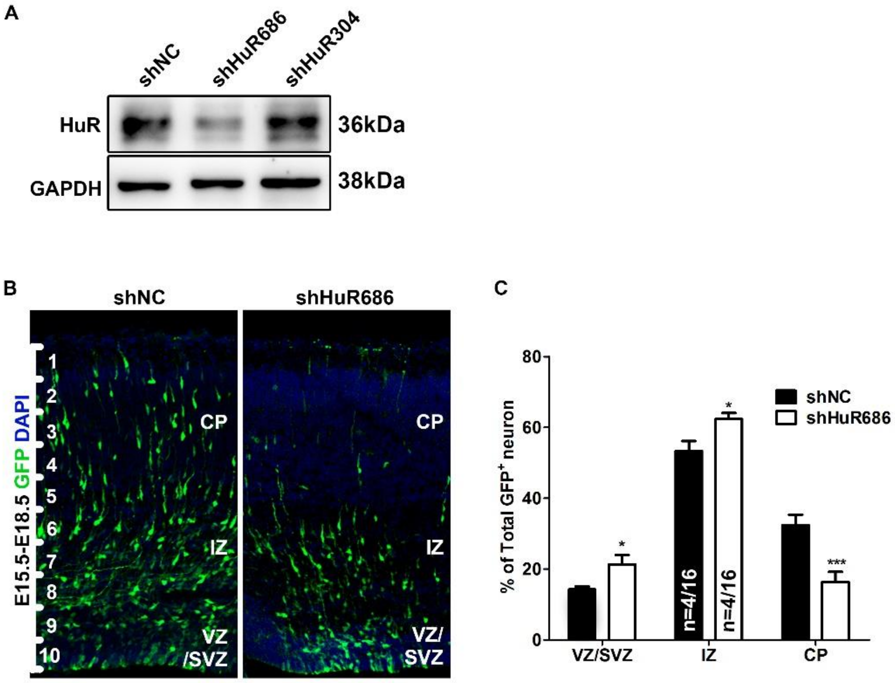

Fig. S3. HuR knockdown impairs neuronal migration. (A) Western blotting analysis showed the knockdown efficiency of shHuR686 was better than that of shHuR304. (B) E15.5 WT embryonic brains were electroporated with indicated plasmids, and cortical slices were stained with an anti-GFP antibody and DAPI at E18.5. Scale bar=100 μm. (C) Cerebral cortices were divided into VZ/SVZ, IZ and

Development • Supplementary information

CP areas. Quantification analyses of GFP^+ cells in each area. Student's t-test was used for statistical analysis. The n values represent the numbers of brains/brain sections. The data are shown as means ± SEM. n.s.: P > 0.5; *: P < 0.05; **: P < 0.01; ***: P < 0.001.

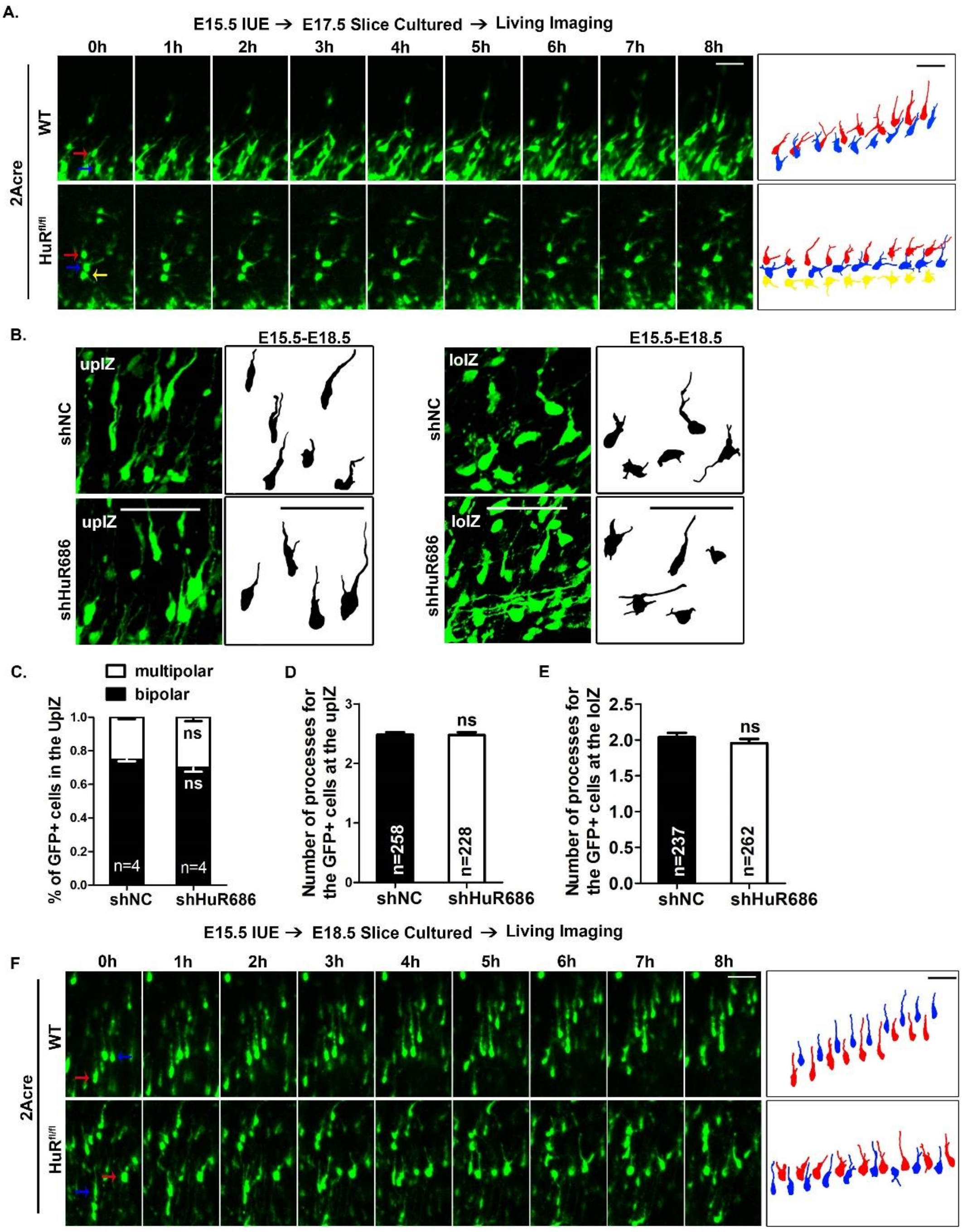

Fig. S4. HuR-deficient neuron exhibits impaired cell motility but normal morphology. (A) Brian slices were obtained 2 days after *in utero* electroporation of

Development • Supplementary information

GFP-2A-Cre plasmids into E15.5 $HuR^{fl/fl}$ or WT embryos. An 8-h time-lapse imaging assay was performed for each slice to acquire serial images of migrating neurons in the IZ. Representative neurons are indicated by red, blue and yellow arrows, and tracings of each neuron are shown in the right panels. Scale bars=20 µm. (B) Representative images of GFP^+ neuron in the upIZ and loIZ of brains that were electroporated with indicated plasmids. Scale bars=20 µm. (C) Quantification of multipolar and bipolar neurons in the upIZ of brains that electroporated with indicated plasmids. Student's t-test was used for statistical analysis. The n values represent the numbers of brains. (D) and (E) Quantification of processes of GFP^+ cells in the upIZ and loIZ. Student's t-test was used for statistical analysis. The n values represent the numbers of neurons. (F) Brain slices were obtained 3 days after *in utero* electroporation of RV-CAG-GFP-2A-Cre plasmids into E15.5 $HuR^{fl/fl}$ or WT embryos. An 8-h time-lapse imaging assay was performed for each slice to acquire serial images of migrating neurons in the CP. Representative neurons are indicated by red and blue arrows, and tracings of each neuron are shown in the right panels. Scale bars= 20 µm. The numbers of brains and neurons quantified in each experiment are indicated on the graphs or shown in the legend. The data are shown as means ± SEM. Student's t-test was used for statistical analyses in this figure. n.s.: $P > 0.5$; *: $P < 0.05$; **: $P < 0.01$; ***: $P < 0.001$.

Development • Supplementary information

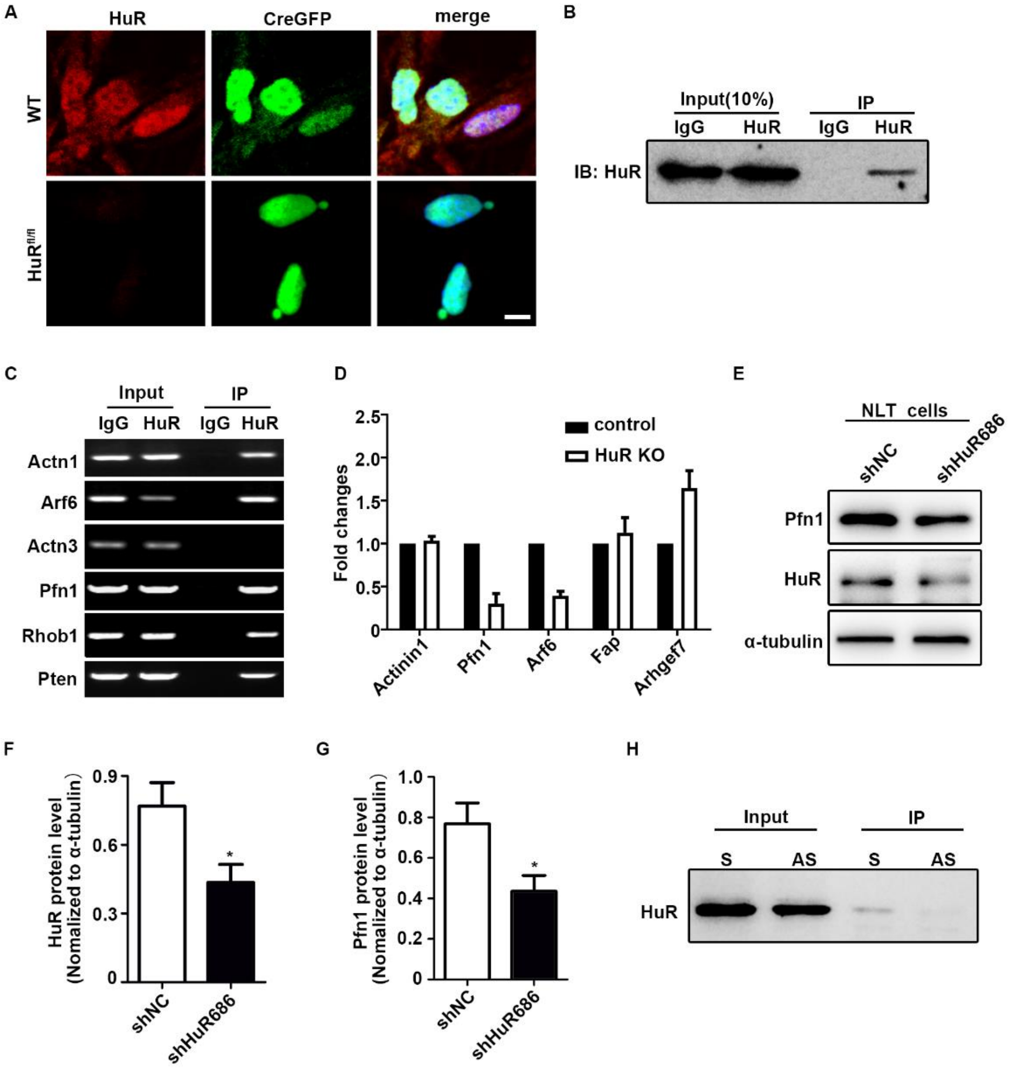

Fig. S5. HuR deficiency impairs Pfn1 expression. (A) Representative images of GFP-Cre lentivirus-infected HuR$^{fl/fl}$ and WT neurons. The absence of HuR staining signal confirmed the deletion of HuR in neurons. (B) RNA-IP using an anti-HuR antibody. (C) RNA-IP of HuR confirmed the binding between HuR protein and the mRNAs of *actn1*, *arf6*, *pfn1*, *rhob1* and *pten*. (D) qPCR analyses of indicated genes that derived from P0 HuR$^{fl/fl}$; Nestin-Cre and HuR$^{fl/fl}$ mice cortical lysates. Mann-Whitney U was used for statistical analysis. Quantification results were from 4 independent experiments. (E) Western blotting analyses of HuR and Pfn1 protein expression in NLT cells that electroporated with shNC or shHuR686 vectors. (F) and (G) Quantitative analyses of HuR (F) and Pfn1 (G) protein expression levels in (E). Quantification results were from

Development • Supplementary information

3 independent experiments. Mann-Whitney U was used for statistical analysis. (H) RNA binding assay of *pfn1* mRNA with HuR protein. The sense RNA template of *pfn1* precipitates with HuR protein. Quantification data are shown as means ±SEM. n.s.: P > 0.5; *: P < 0.05; **: P < 0.01; ***: P < 0.001.

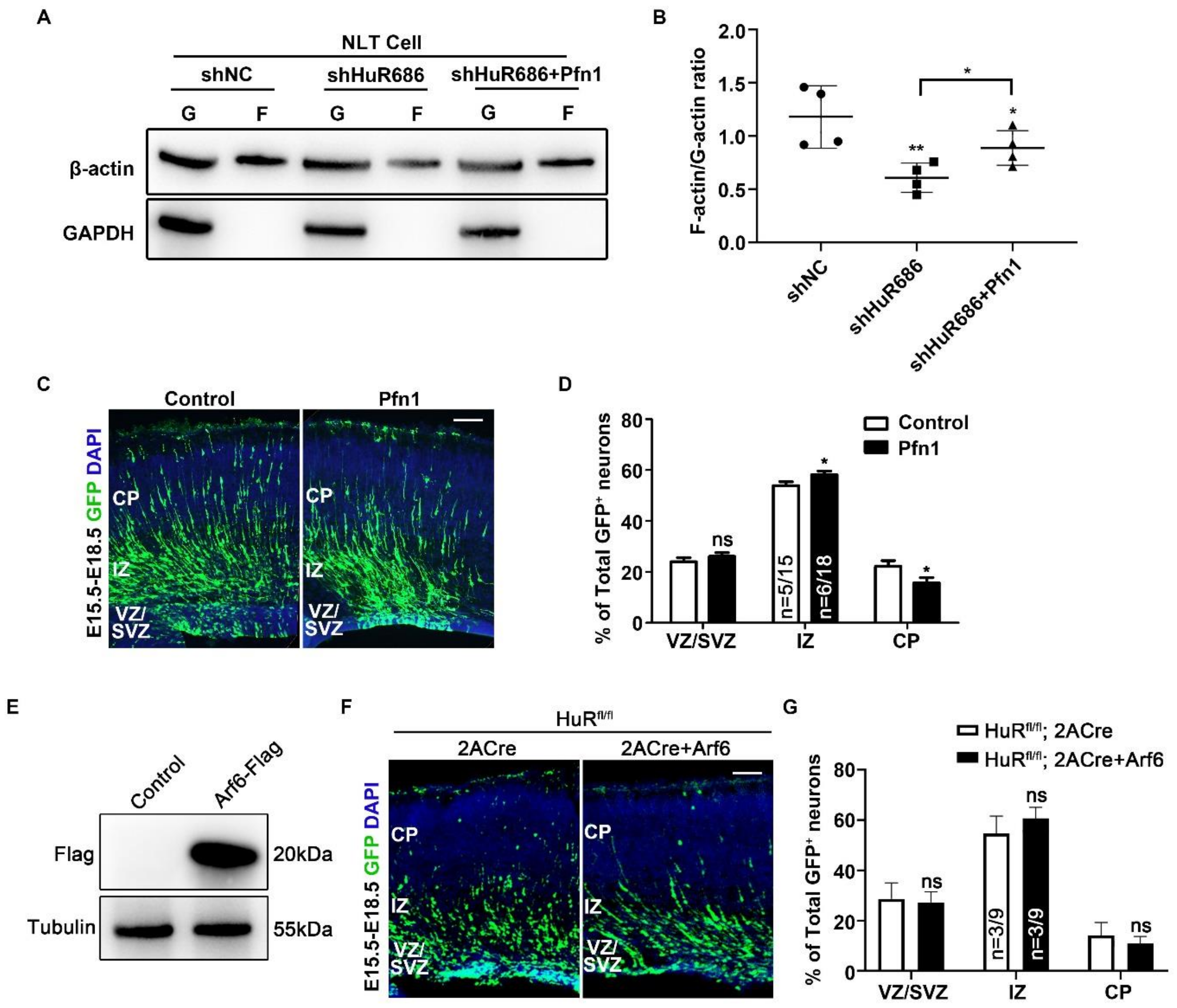

Fig. S6. Pfn1 overexpression rescues migration defects of HuR-deficient neurons. (A) Western blotting analyses of G-actin and F-actin fractions in cultured NLT cells that transfected with indicated plasmid. The mole ratio of shHuR vector to Pfn1 expression vector is 2:1. (B) Quantification of G/F-actin ratio as determined using densitometry analyses of Western blotting results from 4 independent experiments. One-way ANOVA was used for statistical analysis. (C) The control or Pfn1 expression plasmid was electroporated into E15.5 WT embryo cortices. Cortical sections were

Development • Supplementary information

analyzed at E18.5 and immunostained for GFP. The nuclei were counterstained with DAPI. Scale bar=100 µm. (D) Quantification results of GFP^+ neuron distribution across the cerebral cortex. Overexpression Pfn1 alone showed a deleterious effect on neuronal migration. Student's t-test was used for statistical analysis. The n values represent the numbers of brains/brain sections. (E) HEK293T cells were transfected with indicated plasmids. Cell lysates were incubated with an anti-Flag antibody to detect the overexpression of Arf6. β-Tubulin was used as a loading control. (F) E15.5 $HuR^{fl/fl}$ embryonic brains were electroporated with the indicated plasmids, and cortical slices were stained with an anti-GFP antibody and DAPI at E18.5. The mole ratio of 2A-Cre vector to Arf6 expression vector is 1:1. Scale bar=100 µm. (G) Quantification analyses of GFP^+ neuron distribution in (F). Student's t-test was used for statistical analysis. The n values represent the numbers of brains/brain sections. The data are shown as means ±SEM. n.s.: $P > 0.5$; *: $P < 0.05$; **: $P < 0.01$; ***: $P < 0.001$.

Development • Supplementary information

Table S1 Results of PCR-array

Position	Unigene	GeneBank	Description	Gene Name	Fold of Change	Comment
A01	Mm.489605	NM_026064	*Myl12a*	RIKEN cDNA 2900073G15 gene	-1.92	
A02	Mm.403477	NM_134156	*Actn1*	Actinin, alpha 1	-5.84	#
A03	Mm.5316	NM_013456	*Actn3*	Actinin alpha 3	-2.38	#
A04	Mm.81144	NM_021895	*Actn4*	Actinin alpha 4	-1.21	
A05	Mm.259045	NM_146243	*Actr2*	ARP2 actin-related protein 2 homolog (yeast)	-1.46	
A06	Mm.183102	NM_023735	*Actr3*	ARP3 actin-related protein 3 homolog (yeast)	-1.37	
A07	Mm.6645	NM_009652	*Akt1*	Thymoma viral proto-oncogene 1	1.31	
A08	Mm.27308	NM_007481	*Arf6*	ADP-ribosylation factor 6	-3.52	
A09	Mm.474783	NM_133796	*Arhgdia*	Rho GDP dissociation inhibitor (GDI) alpha	-1.55	
A10	Mm.244068	NM_017402	*Arhgef7*	Rho guanine nucleotide exchange factor (GEF7)	3.04	#
A11	Mm.197534	NM_130862	*Baiap2*	Brain-specific angiogenesis inhibitor 1-associated protein 2	-2.22	
A12	Mm.3758	NM_009954	*Bcar1*	Breast cancer anti-	-1.59	

Development • Supplementary information

				estrogen resistance 1		
B01	Mm.6221	NM_007600	*Capn1*	Calpain 1	-2.45	#
B02	Mm.19306	NM_009794	*Capn2*	Calpain 2	-2.00	
B03	Mm.28278	NM_007616	*Cav1*	Caveolin 1, caveolae protein	-2.24	
B04	Mm.447553	NM_009861	*Cdc42*	Cell division cycle 42 homolog (S. cerevisiae)	-2.17	
B05	Mm.329655	NM_007687	*Cfl1*	Cofilin 1, non-muscle	-2.06	
B06	Mm.280125	NM_133656	*Crk*	V-crk sarcoma virus CT10 oncogene homolog (avian)	-1.74	
B07	Mm.795	NM_007778	*Csf1*	Colony stimulating factor 1 (macrophage)	-1.87	#
B08	Mm.490123	NM_007803	*Cttn*	Cortactin	-1.99	#
B09	Mm.195916	NM_007858	*Diap1*	Diaphanous homolog 1 (Drosophila)	-2.26	#
B10	Mm.1151	NM_010074	*Dpp4*	Dipeptidylpeptidase 4	-2.04	#
B11	Mm.252481	NM_010113	*Egf*	Epidermal growth factor	-1.89	#
B12	Mm.420648	NM_007912	*Egfr*	Epidermal growth factor receptor	-1.67	#
C01	Mm.389224	NM_010135	*Enah*	Enabled homolog (Drosophila)	-1.46	
C02	Mm.277812	NM_009510	*Ezr*	Ezrin	-1.44	
C03	Mm.41816	NM_007986	*Fap*	Fibroblast activation protein	-3.10	#

C04	Mm.473689	NM_008006	*Fgf2*	Fibroblast growth factor 2	-2.16	#
C05	Mm.267078	NM_010427	*Hgf*	Hepatocyte growth factor	-2.27	#
C06	Mm.268521	NM_010512	*Igf1*	Insulin-like growth factor 1	-2.29	#
C07	Mm.275742	NM_010513	*Igf1r*	Insulin-like growth factor I receptor	-1.59	
C08	Mm.274846	NM_010562	*Ilk*	Integrin linked kinase	-1.19	
C09	Mm.31903	NM_010576	*Itga4*	Integrin alpha 4	-1.58	#
C10	Mm.263396	NM_010578	*Itgb1*	Integrin beta 1 (fibronectin receptor beta)	-1.69	
C11	Mm.1137	NM_008404	*Itgb2*	Integrin beta 2	-2.49	#
C12	Mm.87150	NM_016780	*Itgb3*	Integrin beta 3	-2.38	#
D01	Mm.15409	NM_010717	*Limk1*	LIM-domain containing, protein kinase	-1.97	#
D02	Mm.196581	NM_011949	*Mapk1*	Mitogen-activated protein kinase 1	-2.06	
D03	Mm.86844	NM_008591	*Met*	Met proto-oncogene	-1.96	#
D04	Mm.486486	NM_008608	*Mmp14*	Matrix metallopeptidase 14 (membrane-inserted)	-1.29	
D05	Mm.29564	NM_008610	*Mmp2*	Matrix metallopeptidase 2	-1.63	#
D06	Mm.4406	NM_013599	*Mmp9*	Matrix	-1.35	#

Development • Supplementary information

				metallopeptidase 9		
D07	Mm.138876	NM_010833	*Msn*	Moesin	-1.42	
D08	Mm.218233	NM_175260	*Myh10*	Myosin, heavy polypeptide 10, non-muscle	-1.39	
D09	Mm.29677	NM_022410	*Myh9*	Myosin, heavy polypeptide 9, non-muscle	-1.53	
D10	Mm.33360	NM_139300	*Mylk*	Myosin, light polypeptide kinase	-1.90	#
D11	Mm.260227	NM_011035	*Pak1*	P21 protein (Cdc42/Rac)-activated kinase 1	-1.58	
D12	Mm.21876	NM_027470	*Pak4*	P21 protein (Cdc42/Rac)-activated kinase 4	-1.69	
E01	Mm.2647	NM_011072	*Pfn1*	Profilin 1	-2.96	
E02	Mm.260521	NM_008839	*Pik3ca*	Phosphatidylinositol 3-kinase, catalytic, alpha polypeptide	-1.76	
E03	Mm.1359	NM_011113	*Plaur*	Plasminogen activator, urokinase receptor	-1.66	#
E04	Mm.44463	NM_021280	*Plcg1*	Phospholipase C, gamma 1	-1.54	
E05	Mm.212039	NM_008875	*Pld1*	Phospholipase D1	-1.44	#
E06	Mm.222178	NM_011101	*Prkca*	Protein kinase C, alpha	-1.50	
E07	Mm.245395	NM_008960	*Pten*	Phosphatase and	1.82	

Development • Supplementary information

				tensin homolog		
E08	Mm.254494	NM_007982	*Ptk2*	PTK2 protein tyrosine kinase 2	-1.31	
E09	Mm.21613	NM_172498	*Ptk2b*	PTK2 protein tyrosine kinase 2 beta	-1.93	#
E10	Mm.277916	NM_011201	*Ptpn1*	Protein tyrosine phosphatase, non-receptor type 1	-1.80	
E11	Mm.18714	NM_011223	*Pxn*	Paxillin	-1.37	#
E12	Mm.469963	NM_009007	*Rac1*	RAS-related C3 botulinum substrate 1	-1.55	
F01	Mm.1972	NM_009008	*Rac2*	RAS-related C3 botulinum substrate 2	-1.92	#
F02	Mm.259653	NM_145452	*Rasa1*	RAS p21 protein activator 1	-1.92	
F03	Mm.472057	NM_009041	*Rdx*	Radixin	-1.50	
F04	Mm.406156	NM_145383	*Rho*	Rhodopsin	-1.87	#
F05	Mm.318359	NM_016802	*Rhoa*	Ras homolog gene family, member A	-1.66	
F06	Mm.687	NM_007483	*Rhob*	Ras homolog gene family, member B	1.33	
F07	Mm.262	NM_007484	*Rhoc*	Ras homolog gene family, member C	-1.65	
F08	Mm.46497	NM_028810	*Rnd3*	Rho family GTPase 3	-1.82	
F09	Mm.6710	NM_009071	*Rock1*	Rho-associated coiled-coil containing protein kinase 1	-2.04	
F10	Mm.127560	NM_008018	*Sh3pxd2a*	SH3 and PX domains 2A	-1.63	#

Development • Supplementary information

F11	Mm.22845	NM_009271	*Src*	Rous sarcoma oncogene	-1.79	#
F12	Mm.249934	NM_011486	*Stat3*	Signal transducer and activator of transcription 3	-1.71	
G01	Mm.136791	NM_153153	*Svil*	Supervillin	-1.78	
G02	Mm.248380	NM_011577	*Tgfb1*	Transforming growth factor, beta 1	-2.51	#
G03	Mm.206505	NM_011594	*Timp2*	Tissue inhibitor of metalloproteinase 2	-2.19	
G04	Mm.208601	NM_011602	*Tln1*	Talin 1	-2.08	
G05	Mm.9684	NM_009499	*Vasp*	Vasodilator-stimulated phosphoprotein	-1.87	#
G06	Mm.279361	NM_009502	*Vcl*	Vinculin	-2.15	
G07	Mm.282184	NM_009505	*Vegfa*	Vascular endothelial growth factor A	-1.73	
G08	Mm.268000	NM_011701	*Vim*	Vimentin	-1.98	
G09	Mm.41353	NM_031877	*Wasf1*	WASP family 1	-1.59	
G10	Mm.23566	NM_153423	*Wasf2*	WAS protein family, member 2	-1.76	#
G11	Mm.1574	NM_028459	*Wasl*	Wiskott-Aldrich syndrome-like (human)	-1.74	
G12	Mm.223504	NM_153138	*Wipf1*	WAS/WASL interacting protein family, member 1	-2.14	#
H03	Mm.304088	NM_008084	*Gapdh*	Glyceraldehyde-3-	1.44	internal

Development • Supplementary information

				phosphate dehydrogenase		control
H05	Mm.2180	NM_008302	*Hsp90ab1*	Heat shock protein 90 alpha (cytosolic), class B member 1	-1.44	Internal control

Gene expression was analyzed by Qiagen online software (https://www.qiagen.com/cn/shop/genes-and-pathways/data-analysis-center-overview-page/) using Gapdh and Hsp90ab1 as internal control. Up-regualted genes were shown as positive values and down-regulated genes were shown as negative values. # indicated genes that had low basal expression level (Ct value>30 in control samples). The most up-regulated gene (*Arhgef7*) was colored in RED, and the most down-regulated genes (*Actn1*, *Arf6*, *Fap*, *Pfn1*) were colored in GREEN.

Development • Supplementary information

Table S2 Sequences of primers in this study

**Primers**	**Sequence 5' to 3'**	**Notes**
NEX Cre 1:	CCGCATAACCAGTGAAACAG	(Goebbels et al., 2006)
NEX Cre 2:	AGAATGTGGAGTAGGGTGAC	Pair with NEX Cre 1
NEX Cre 3:	GAGTCCTGGCAGTCTTTTTC	Pair with NEX Cre 1
HuR flox F:	CTCTCCAGGCAGATGAGCA	(Ghosh et al., 2009)
HuR flox R:	TAGGCTCTGGGATGAAACCT	
Ai27D Wild F:	AAG GGA GCT GCA GTG GAG TA	(Linda et al., 2012)
Ai27D Wild R:	CCGAAAATCTGTGGGAAGTC	
Ai27D Mutant F:	CTGTTCCTGTACGGCATGG	(Linda et al., 2012)
Ai27D Mutant R:	GGCATTAAAGCAGCGTATCC	
Nestin-Cre 1:	GCCTTATTGTGGAAGGAC	(Giusti et al., 2014)
Nestin-Cre 2:	TTGCTAAAGCGCTACATAGGA	Pair with Nestin-Cre 1
Nestin-Cre 3	CCTTCCTGAAGCAGTAGAGCA	Pair with Nestin-Cre 1
1-81 3'UTR: F:	CCGCTCGAGCCTCATCTGTCCCTTCCCC CCACCG	Pfn1 cloning
1-81 3'UTR: R:	TTTTCCTTTTGCGGCCGCAAAAAATAAT GGTATGTGTG	Pfn1 cloning
82-290 3'UTR: F:	CCGCTCGAGGGGCCATTACCCCATTTC	Pfn1 cloning
82-290 3'UTR: R:	TTTTCCTTTTGCGGCCGCTTTTTTTTTTT TTTTGTTAG	Pfn1 cloning
1-194 3'UTR: R:	TTTTCCTTTTGCGGCCGCTTTTCCAAACACACACAG GA	Pfn1 cloning
HuR F:	TTCTGGTGTCAATGTCCCCG	qPCR
HuR R:	CAAAGGGGCCAAACATCTGC	qPCR
GAPDH F:	GAGAGACCCTCACTGCTG	qPCR
GAPDH R:	GATGGTACATGACAAGGTGC	qPCR
Profilin1 F:	GTGGAACGCCTACATCGACA	qPCR

Development • Supplementary information

Profilin1 R：	TTGACCGGTCTTTGCCTACC	qPCR
Actn1 F：	ACATGCAGCCTGAAGAGGAC	qPCR
Actn1 R：	TGAGATGACCTCCAGGAGCA	qPCR
Arf6 F：	ATCTTCGCCAACAAGCAGGA	qPCR
Arf6 R：	AGGTTAACCATGTGAGCCCC	qPCR
Actn3 F：	GAGCTCGACTACCATGAGGC	qPCR
Actn3 R：	GCCAGTTATTGAAGGGGGCT	qPCR
Rhob F：	ACTATGTGGCGGACATCGAG	qPCR
Rhob R：	AGAAGTGCTTTACCTCGGGC	qPCR
Pten F：	TCCTGCAGAAAGACTTGAAGGT	qPCR
Pten R：	GCTGTGGTGGGTTATGGTCT	qPCR
Fap F:	GGCTGGGGCTAAGAATCCG	qPCR
Fap R:	GCATACTCGTTCACTGGACAC	qPCR
Arhgef7 F:	CATAATCACGTCTTGGCTGATGA	qPCR
Arhgef7 R	CAGCAGCTCTTACGGGATGC	qPCR
pfn1-F	CCTCATCTGTCCCTTCCCCCCACCG	RNA binding assay
pfn1-R	GTTAGTAGAATCTTTTTTTATTCAGAAAAA	RNA binding assay

Development • Supplementary information

References

Ghosh, M., Aguila, H. L., Michaud, J., Ai, Y., Wu, M.-T., Hemmes, A., Ristimaki, A., Guo, C., Furneaux, H. and Hla, T. (2009). Essential role of the RNA-binding protein HuR in progenitor cell survival in mice. *J. Clin. Investig.* **119**, 3530-3543. doi:10.1172/JCI38263

Giusti, S. A., Vercelli, C. A., Vogl, A. M., Kolarz, A. W., Pino, N. S., Deussing, J. M. and Refojo, D. (2014). Behavioral phenotyping of Nestin-Cre mice: implications for genetic mouse models of psychiatric disorders. *J. Psychiatr. Res.* **55**, 87-95. doi:10.1016/j.jpsychires.2014.04.002

Goebbels, S., Bormuth, I., Bode, U., Hermanson, O., Schwab, M. H. and Nave, K.-A. (2006). Genetic targeting of principal neurons in neocortex and hippocampus of NEX-Cre mice. *Genesis* **44**, 611-621. doi:10.1002/dvg.20256

Linda, M., Tianyi, M., Henner, K., Jia-Min, Z., Antal, B., Shigeyoshi, F., Hsu, Y. W. A., Garcia, A. J., Xuan, G. and Sebastien, Z. (2012). A toolbox of Credependent optogenetic transgenic mice for light-induced activation and silencing. *Nat. Neurosci.* **15**, 793. doi:10.1038/nn.3078
