# Supplementary material for: Human antigen R-regulated mRNA metabolism promotes the cell motility of migrating mouse neurons
Source: Development. 2020 Mar 16;147(6):dev183509. doi: 10.1242/dev.183509 (PMC7097226; doi:10.1242/dev.183509)
Supplement: Reviewer comments [file develop-147-183509_review_history.pdf]

Development

Human antigen R-regulated mRNA metabolism promotes the cell motility of migrating neurons

Yi-Fei Zhao, Xiao-Xiao He, Zi-Fei Song, Ye Guo, Yan-Ning Zhang, Hua-Li Yu, Zi-Xuan He, Wen-Cheng Xiong, Weixiang Guo and Xiao-Juan Zhu
DOI: 10.1242/dev.183509

Editor: Francois Guillemot

Review timeline

Original submission:	7 August 2019
Editorial decision:	23 September 2019
First revision received:	16 January 2020
Editorial decision:	10 February 2020
Second revision received:	11 February 2020
Accepted:	13 February 2020

Original submission

First decision letter

MS ID#: DEVELOP/2019/183509

MS TITLE: Human Antigen R regulated mRNA metabolism is essential for neuronal migration during neocortex development

AUTHORS: Xiaoxiao He, Yifei Zhao, Yanning Zhang, Ye Guo, Huali Yu, Zixuan He, Wencheng Xiong, Weixiang Guo, and Xiaojuan Zhu

I have now received the reports of three referees on your manuscript and I have reached a decision. The referees' comments are appended below, and you can access them online: please go to BenchPressand click on the 'Manuscripts with Decisions' queue in the Author Area.

As you will see, all the referees express significant interest in your work, but they also criticise multiple aspects of the study and recommend an extensive revision of your manuscript before we can consider publication. In particular, they request that you study whether additional HuR targets than Profilin1 might also contribute to HuR functionin cortical neuron migration. They also ask that you describe better your quantification and statistical methods, that you examine progenitor proliferation more directly using EdU incorporation and/or proliferation markers, and that you cite better previous relevant studies.
If you are able to revise the manuscript along the lines suggested by the different referees, which may involve further experiments, I will be happy to receive a revised version of the manuscript. Your revised paper will be re-reviewed by the original referees, and its acceptance will depend on your addressing satisfactorily all their major concerns. Please also note that Development will normally permit only one round of major revision.

Please attend to all of the reviewers' comments and ensure that you clearly highlight all changes made in the revised manuscript. Please avoid using 'Tracked changes' in Word files as these are lost in PDF conversion. I should be grateful if you would also provide a point-by-point response detailing how you have dealt with the points raised by the reviewers in the 'Response to Reviewers' box. If you do not agree with any of their criticisms or suggestions please explain clearly why this is so.

© 2020. Published by The Company of Biologists under the terms of the Creative Commons Attribution License (https://creativecommons.org/licenses/by/4.0/).

Reviewer 1

Advance summary and potential significance to field

The function of HuR in post mitotic neurons was studied using Cre mediated deletion. It has been demonstrated that HuR deletion affects the motility of post mitotic neurons. It is suggested that a possible down-stream target of HuR, Profiiln 1 (Pfn1), is regulating this effect and over expression of Pfn1 rescues the observed phenotype.

Comments for the author

The manuscript requires extensive and detailed English editing.
The title is very wide and not suitable for the current study.

The authors should refer to and compare their studies with T. Popovitchenko et al. Sci Rep. 2016; 6: 28998. doi: 10.1038/srep28998. What is the overlap with their study? What are the differences?

Direct evidence that HuR binds and stabilises Pfn1 is limited. These studies need to be expanded using additional methods.

Not always the most appropriate experiments were done to demonstrate a particular point.
For example the authors concluded that there is no change in the proliferation of the progenitors based on measurements of the GFP-progenitor marker positive cells three days after in utero electroporation (supplementary figure 2). To investigate this issue, the authors need to look one day after the electroporation of Cre, introduce EdU or BrdU label and immunostain with different progenitor markers. At E18.5 a very small percent of the cells remain in cell cycle.

The morphology of the migrating cells. From the images in Fig. 3, it is very difficult to deduce the structure of the migrating neurons. To enable such a study, membrane-bound GFP should be used combined with sparse labelling. The cells highlighted in A are most likely multipolar. It is possible that the mutant cells spend more time in the multipolar stage, this has not been investigated. Thus, there could be additional effects that result in a shorter migration distance.

The observed effect on actin polymerisation could be indirect.

Statistics and figures.
The authors should indicate in each figure section where they have used ANOVA and where they have used t-test. It is unclear why a different number of sections were used for the different genotypes and if the sections were matched. For example, in case of Fig. 1 G, ANOVA should be used and not t-test since these are several comparisons from different areas of the same brains. The same number of sections should be used for the different brains, unclear why there is a different number of sections per brain. In addition as there is a gradient of brain development (unclear if coronal or saggital sections were used), the different sections that are compared should be matched. In case of Fig. 1 H, perhaps matched t-test can be appropriate. In is not mentioned how many cells were counted or what area was counted.

Figure 2 and other figures, it is mentioned that the number of brains, brain sections and neurons quantified are presented, however the number of neurons is not presented in any of the figures. Fig. 2 C,D one-way ANOVA. Fig. 2 H two way ANOVA.

Fig. 5 A-B, it should be noted that the effect of HuR on F-actin dynamics could be very indirect and not necessarily through Pfn1.

Reviewer 2

Advance summary and potential significance to field

The manuscript by Zhao et al describes the roles of Human Antigen R (HuR) in cortical neuronal migration in mice. The authors showed that neuron-specific conditional deletion of HuR disturbed

© 2020. Published by The Company of Biologists under the terms of the Creative Commons Attribution License (https://creativecommons.org/licenses/by/4.0/).

cortical lamination and neuronal migration. They also showed that HuR deficiency decreased profilin1 mRNA expression. Expression of profilin1 in the HuR-knockout neurons partially restored the neuronal migration defects.
This study indicates that mRNA metabolism plays an important role in neuronal migration. However, this reviewer finds two major problems. First, previous studies have already showed the involvement of HuR and profilin1 in cortical lamination and neuronal migration, respectively (PNAS, 2014, 111(36):E3815-E3824. EMBO R 2012, 13(1):75-82), which reduces a novelty in this manuscript. Second, although the rescue experiments are important in this manuscript, profilin expression may not be enough to restore the migration defects of the HuR-deficient neurons.
Overall, this study is a potentially important, but additional efforts to identify a functionally important downstream mRNA of HuR are required.

Comments for the author
[Major points]
1) The HuR-deficient neurons are accumulated in the IZ and VZ/SVZ. However, HuR expression is hardly observed in the IZ and VZ/SVZ at E15.5 (Fig. 1D). The authors should explain the inconsistent data. In addition, both multipolar and bipolar neurons are located at the IZ. It is needed to clarify which type of the migrating neurons expresses HuR.
2) The authors also mentioned that HuR is expressed in the CDP-positive neurons. However, the expression of HuR is weak in the CDP-positive upper layers at E18.5 (Fig. 1D). These images may indicate that HuR is expressed in the mature deep layer neurons (but less expressed in the migrating and upper layer neurons).
3) As I mentioned above, profilin expression barely rescued the migration defects of the HuR-deficient neurons. The migration distance of the HuR-deficient neurons is 30-40 um (Fig. 3E), but the profilin1-electroporated neurons is around 20 um (Fig. 5G), suggesting that profilin1 could not rescue the migration defects. The authors should examine whether other downstream candidates, such as Arp2/3, cofilin and Rnd3 (from Table S1), rescue the phenotypes of the HuR-deficient neurons or not.
4) The HuR-deficient bipolar neurons seem to have an abnormally branched leading process. A recent report indicates that knockdown of Cav1, a HuR target gene candidate (Table S1), results in branched leading processes (iScience, 2018, 7:53-67). Because profilin expression may not rescue the branching phenotype, it would be better to analyze whether Cav1 is another functional target of HuR in the regulation of proper neuronal morphologies.
5) HuR deletion reduces the ratio of F-actin to total actin. The authors should examine whether profilin1 expression rescues this phenotype.
6) In Fig. 2H, the MAP2-positive cells were counted. Because MAP2 is a marker for mature neurons that finish the neuronal migration, the expression of MAP2 in the migrating neurons should be very low. It is needed to provide the high magnification images and the single color (red and green) images. Are the expression levels of MAP2 in the migrating neurons comparable with that of the surrounding mature neurons?

[Minor points]
7) The authors mentioned that HuR deficiency disturbed neuronal polarity (Fig. 3G). Do they analyze the position of centrosome or golgi? If not, the authors should weaken the statements.
8) Profilin1 is known to control actin dynamics in cooperation with other actin-binding proteins, such as cofilin and Arp2/3. Table S1 indicates that HuR regulates the mRNA expression of several other actin-binding proteins (e.g. Arp2/3 cofilin, actinin1 and myosin-10) and actin-regulatory proteins (e.g. Rnd3, Rac1 and Cdc42). Among them, Arp2/3 (Development, 2016, 143(15):2741-2752), cofilin (Nat Cell Biol, 2006, 8(1): 17-26), myosin-10 (Cerebral Cortex, 2014 24(5):1259-1268), Rnd3 (Neuron, 2011, 69(6):1069-1084), Rac1 (EMBO J, 2003, 22(16): 4190-4201. Cell Rep, 2012 2(3):640-651) and Cdc42 (J Biol Chem, 2005, 280(6):5082-5088. Nat Neurosci, 2006, 9(1):50-57) have been shown to regulate neuronal migration. It would be better to discuss the relationship between profilin1 and these molecules in neuronal migration.

Reviewer 3

Advance summary and potential significance to field

The manuscript “Human Antigen R regulated mRNA metabolism is essential for neuronal migration during neocortex development” describes the function of the HuR proteins in neuronal migration

© 2020. Published by The Company of Biologists under the terms of the Creative Commons Attribution License (https://creativecommons.org/licenses/by/4.0/).

during corticogenesis. The authors analyzed the function of HuR proteins in cortical development and radial neuronal migration. They found that HuR proteins are expressed in the developing cortical plate, especially in post-mitotic neurons. Genetical deletion of HuR gene in post-mitotic neurons using NEX-Cre affected the thickness of CDP-positive late-born neuron layer. They demonstrated that HuR proteins are important for normal radial migration of late-born neurons by BrdU injection and IUE experiments. Then they performed time-lapse imaging of HuR depleted neurons and found that migration distances were decreased compared to the control neurons. Using cell motility PCR array, they have screened for binding partners for HuR and found that profilin 1 is a strong candidate. So, they examined if HuR can stabilize mRNA of Profilin 1 and found that HuR is critical for mRNA stability of profilin 1. Finally, they indicated that Profilin 1 can rescue the migration defect phenotypes of HuR depleted neurons in terms of their migration distance and cell motility from which they lead to the conclusion that HuR is required to promote the cell motility via stabilization of profilin 1 mRNA during radial migration process.

This study provides interesting insights into HuR-Pfn1 dependent regulatory mechanism of radial neuronal migration during corticogenesis. However, several points need to be addressed prior publication in order to support the authors' conclusions:

Comments for the author

1. Fig 1 A-D
The authors claim that HuR proteins are localized in projection neurons in cortical plate because projection neurons were the major cell type in the cortical plate (line 80). However, GABA-positive inhibitory neurons should also be in the cortex although the cell number is low in this embryonic stage. They should check if GABAergic neurons have HuR proteins in the different embryonic or postnatal stages to reach their conclusion.

2. Fig S2
They claim that HuR deletion did not affect neural progenitor cell proliferation and differentiation by immunostaining of PAX6, Tbr2 and Ki67. These pictures in Fig S2-A, there are not so many GFP positive neurons in the ventricular zone compared to Fig 2E. They should show the whole sections of Fig S2A in supplemental data.

3. Fig 3
There is only one trace of migrating cell in each live imaging. They should show more several traces for each condition as supplemental data. 8hrs time-lapse imaging is not sufficient for observing the migration status. Can they demonstrate any longer imaging data? Moreover, they didn't describe how to calculate the migration distances from the movie data in the method section. They should specify the method including the name of the analyzing software. If they didn't use any software, they should show the several examples of hand tracing lines they measured. About the graph G, they also should mention how to measure the % of "loosing polarity". About the numbers of measured samples, the definition is not clear. They should state the detailed brain number or slice number and how many times of the same independent experiments.

4. The reason they chose pfn1 as a candidate for binding partner of HuR was not clear. According to their gene expression data of cell motility PCR array, I found that Actn1, Arf6 and Arhgef 7 are highly downregulated or upregulated compared to pfn1. All of them are related to actin dynamics and cell motility, suggesting the possibility that they also contributed to the phenotype shown in HuR knockout neurons. How they chose only pfn1 to further analysis is not clear to me. Please mention about this point in the manuscript.

5. Fig4
Pfn1 mRNA stability is decreased when HuR proteins are depleted (F). Is there any mRNA of which the stability is not affected by HuR depletion? They should indicate this example as a negative control.

6. Fig 5
Again, they should show more examples of cell traces for migrating behavior as a supplemental data.

© 2020. Published by The Company of Biologists under the terms of the Creative Commons Attribution License (https://creativecommons.org/licenses/by/4.0/).

7. They claim that the MP-BP transition is not impaired but the motilities of bipolar shape is decreased. However, it’s hard to convince people from the present data. Please show other pictures so that they can see the author’s claim.

Minor points
1. In the introduction part, you cited Ayala et al., 2007 to explain the radial migration process. However, there are more recent review such as “Ohtaka-Maruyama and Okado, Frontiers in Neuroscience, 2015 Dec 17;9:447.2018” is also recommended.

2. The reason for that the migration of later-born neurons are more affected than deep layer neurons although HuR is expressed in the both cell population should be discussed in the discussion part.

3. About the rescue experiment of HuR deleted neurons by Pfn1, the authors should try several different concentrations of Pfn1 expression plasmids because the overexpression of Pfn1 impairs the radial migration (Fig.S4B). In this regards, they should specify the concentration of rescue plasmid concentration in the method part instead of describing “in designed dosage” (line315).

First revision

Author response to reviewers' comments

Reviewer 1 Advance Summary and Potential Significance to Field:
The function of HuR in post mitotic neurons was studied using Cre mediated deletion. It has been demonstrated that HuR deletion affects the motility of post mitotic neurons. It is suggested that a possible down-stream target of HuR, Profiiln 1 (Pfn1), is regulating this effect and over expression of Pfn1 rescues the observed phenotype.

Reviewer 1 Comments for the Author:
The manuscript requires extensive and detailed English editing.
Response: To address the language issue, the revised manuscript has been edited by professional English editing service (www.AJE.com). Corrections were made throughout the manuscript.

The title is very wide and not suitable for the current study.
Response: The title of the revised manuscript has been amended to “Human Antigen R regulated mRNA metabolism promotes the cell motility of migrating neurons”.

The authors should refer to and compare their studies with T. Popovitchenko et al. Sci Rep. 2016; 6: 28998. doi: 10.1038/srep28998. What is the overlap with their study? What are the differences?
Response: We thank the review to point out this reference, which we missed in our previous manuscript. We have carefully read and studied the data in this paper. In their work, Popovitchenko and colleagues identified HuR binding partners in neocortices at different developmental stages. Then, they focused on how HuR differentially regulated Foxp1 and Foxp2 mRNAs. Although Foxp1 deficiency was previously shown to regulate neuronal migration (Li et al., PLoS One. 2015; 10(5): e0127671), but data in Popovitchenko’s paper showed HuR knockout increased Foxp1 protein level rather than decreased Foxp1 protein level. Therefore, results in Popovitchenko’s paper did not indicate whether HuR plays a role in neuronal migration and what would be the underlying mechanism.
In our current manuscript, we focused on how HuR regulate migrating neurons and what are the downstream effectors of HuR in such regulation. Although the mice models were similar in these two studies, we used total different technique sets to study the function of HuR. We think there is minimal overlapping between these two studies. However, as an important earlier study that showed HuR functions during neocortex development. This paper was added in our revised manuscript. See lines 62 and 83.

Direct evidence that HuR binds and stabilises Pfn1 is limited. These studies need to be expanded

© 2020. Published by The Company of Biologists under the terms of the Creative Commons Attribution License (https://creativecommons.org/licenses/by/4.0/).

using additional methods.
Response: In our previous manuscript, we provided several lines of evidence to show HuR binds with Pfn1 mRNA and regulates Pfn1 expression. We firstly showed HuR binds with *pfn1* mRNA using RNA-IP and then validated the direct binding of HuR with *pfn1* mRNA 3' UTR using psiCHECK2 system. Data from these two experiments showed HuR can directly bind with Pfn1 mRNA. Then we studied the *pfn1* mRNA degradation rate and Pfn1 protein levels in HuR deficient cells. Our results showed HuR positively regulated Pfn1 mRNA and protein levels.
During the revision of this manuscript, we performed RNA binding assay to test if *pfn1* mRNA could bind with HuR protein. Result showed the sense *pfn1* mRNA but not the antisense *pfn1* mRNA could bind with HuR protein. This result further validated our previous result that HuR could bind with pfn1 mRNA. For detail of this result, please see lines197-198 and Fig. S5H.

Not always the most appropriate experiments were done to demonstrate a particular point. For example the authors concluded that there is no change in the proliferation of the progenitors based on measurements of the GFP-progenitor marker positive cells three days after in utero electroporation (supplementary figure 2). To investigate this issue, the authors need to look one day after the electroporation of Cre, introduce EdU or BrdU label and immunostain with different progenitor markers. At E18.5 a very small percent of the cells remain in cell cycle.
Response: We agree E18.5 was not a suitable time window to study proliferation and differentiation of neural progenitor cells. During the revision of this manuscript, a paper was published from our collaborator (Dr. Weixiang Guo, who is also a correspondence author of this manuscript). In their work, they studied the functions of HuR in neurogenesis during embryonic and adult stages. Their results showed that HuR conditional deletion by EMX1- Cre did not affect embryonic neurogenesis (Wang et al., 2019, Cell Reports, DOI:10.1016/j.celrep.2019.10.127).

To further clarify if HuR deletion has any effect on the fate of neural progenitor cells, we performed independent experiments using HuR$^{fl/fl}$; Nestin-Cre mice, which deleted HuR in all types of neural progenitor cells. BrdU was injected at E14.5 and brain slices were prepared in 2 h and 48 h, respectively. Results showed the ratios of PAX6+ or Tbr2+ cell among total BrdU+ cell were similar between HuR$^{fl/fl}$; Nestin-Cre and HuR$^{fl/fl}$ mice in 2 h post BrdU injection. And the ratio of Tbr1+ cell among total BrdU+ cell was also similar between HuR$^{fl/fl}$; Nestin-Cre and HuR$^{fl/fl}$ mice in 48 h post BrdU injection. Altogether, our new data and results from Dr. Guo's recent publication revealed that HuR did not impair neurogenesis during embryonic development. For detail of these results please see lines 86-94 and Fig.S1.

The morphology of the migrating cells. From the images in Fig. 3, it is very difficult to deduce the structure of the migrating neurons. To enable such a study, membrane-bound GFP should be used combined with sparse labelling. The cells highlighted in A are most likely multipolar. It is possible that the mutant cells spend more time in the multipolar stage, this has not been investigated. Thus, there could be additional effects that result in a shorter migration distance.
Response: In our experiment, the Cre-GFP vector restricted GFP expression in nuclei, which cannot be used for morphology analysis. And the time-lapse images were also not ideal for such analysis. To address this issue, we added an additional data set, in which HuR knockdown was performed in migrating neurons. The GFP signal in the pGeneClip™ hMGFP backbone showed cytoplasmic distribution, which is suitable for morphology analysis of the migrating neurons. Results from HuR knockdown assay revealed that the multipolar-to-bipolar transition in the IZ was not impaired. But the neuronal migration defects maintained. We think this new data could support our conclusion that the cell motility defect rather than the multipolar-to-bipolar transition defect was the major causal factor to delay neuronal migration. Please find these new data in lines 161-165 and Fig. S4B-E.

The observed effect on actin polymerisation could be indirect.
Response: We thank the reviewer to point out this issue. To answer this question, we tried to rescue the actin polymerization in HuR deficient NLT cells via co-expression with Pfn1. We found the ratio of F-actin fraction was decreased in HuR knockdown NLT cells. And the ratio of F-actin fraction was significantly elevated when Pfn1 was co-expressed with shHuR plasmid. Therefore, we conclude that the actin polymerization in HuR deficient cells could be rescued by Pfn1 overexpression. Please find these new results in lines 229-232 and Fig. S6A-B.

Statistics and figures.

© 2020. Published by The Company of Biologists under the terms of the Creative Commons Attribution License (https://creativecommons.org/licenses/by/4.0/).

The authors should indicate in each figure section where they have used ANOVA and where they have used t-test. It is unclear why a different number of sections were used for the different genotypes and if the sections were matched. For example, in case of Fig. 1 G, ANOVA should be used and not t-test since these are several comparisons from different areas of the same brains. The same number of sections should be used for the different brains, unclear why there is a different number of sections per brain. In addition as there is a gradient of brain development (unclear if coronal or saggital sections were used), the different sections that are compared should be matched. In case of Fig. 1 H, perhaps matched t-test can be appropriate. In is not mentioned how many cells were counted or what area was counted.

Response:
As the reviewer suggested, we have indicated the statistical method in the figure legend for each individual quantification result.

For Fig.1G (now Fig.1D in revised manuscript), reviewer raised several questions regarding the number of sections, coronal or saggital sections, if the sections are matched positions and how many cells were counted. In our test, we aimed to compare the CDP+ cells distribution in the primary somatosensory cortex (Bregma -0.82 to -1.34). Coronal sections within this area were used for cell counting. Therefore, we did not select exactly the same sections numbers for quantification analysis. However, as the reviewer suggested, we re-selected the brain sections that localized at comparable positions (Bregma -0.82 to -1.06). Four sections per brain were used for cell counting. In each section, a 200 μm wide cortical area was subjected to cell counting. All CDP+ cells within this area were counted. The regular cell number in such area is around 150-300 cells per section. We have added more details of this strategy in the method part. Please see lines 553-556.

Regarding statistical method for Fig. 1 G (now Fig. 1E in revised manuscript), we used t- test to compare the thickness of each area between the WT and HuR KO groups. We did not change the statistical analysis to ANOVA, because the thickness data of different cortical areas were obtained independently. In that case, we did not consider the cortical area as a variable in the analysis. We think this is common in similar studies. If the reviewer still has concern regarding to this issue. We may separate the figure to show these data individually.

For Fig.1 F (previously Fig. 1H), Fig.2 C&D (mentioned in next question), Fig.S2 E&F we have changed the statistical analysis method to Two-way ANOVA with Bonferroni post hoc analysis. Genotype and cortical area were considered as two variables in these analyses.

Figure 2 and other figures, it is mentioned that the number of brains, brain sections and neurons quantified are presented, however the number of neurons is not presented in any of the figures. Fig. 2 C,D one-way ANOVA. Fig. 2 H two way ANOVA.
Response:
We are sorry for this confusion. In figure 2, the data were all based on brain sections. Therefore, we only labeled the numbers of brains and brain sections in the figure. In figure 3B and E, data were obtained from individual neuron. Thus, the n value represent the numbers of brains and neurons. To avoid such confusion, we have added the n value information in the figure legend to specify the meaning. Please see the revised figure legend.

For Fig. 2C and D, we re-performed statistical analysis. Because the genotype and the position of cortical area were considered as two variables, we performed two-way ANOVA with Bonferroni post hoc analysis for statistical analysis. This information is added in the figure legend and method part. See lines 566-567.

For Fig. 2H, we used one-way ANOVA. We just want to compare the GFP+ cells that have already entered the MAP2+ zone. So it is to compare the means between three electroporated groups. The labeling of Y axis was somehow misleading. We have changed the labeling of Y axis to “% of GFP+ cells in the MAP2+ area”.

Fig. 5 A-B, it should be noted that the effect of HuR on F-actin dynamics could be very indirect and not necessarily through Pfn1.
Response: We tried to figure out the relationship between HuR mediated F-actin polymerization and

© 2020. Published by The Company of Biologists under the terms of the Creative Commons Attribution License (https://creativecommons.org/licenses/by/4.0/).

Pfn1 in NLT cells. We have explained the detail of this new data in an earlier question. These new results were added to the revised manuscript. Please see lines 229-232 and Fig. S6A-B.

Reviewer 2 Advance Summary and Potential Significance to Field:
The manuscript by Zhao et al describes the roles of Human Antigen R (HuR) in cortical neuronal migration in mice. The authors showed that neuron-specific conditional deletion of HuR disturbed cortical lamination and neuronal migration. They also showed that HuR deficiency decreased profilin1 mRNA expression. Expression of profilin1 in the HuR- knockout neurons partially restored the neuronal migration defects.This study indicates that mRNA metabolism plays an important role in neuronal migration.
However, this reviewer finds two major problems. First, previous studies have already showed the involvement of HuR and profilin1 in cortical lamination and neuronal migration, respectively (PNAS, 2014, 111(36):E3815-E3824. EMBO R, 2012, 13(1):75-82), which reduces a novelty in this manuscript. Second, although the rescue experiments are important in this manuscript, profilin expression may not be enough to restore the migration defects of the HuR-deficient neurons.
Overall, this study is a potentially important, but additional efforts to identify a functionally important downstream mRNA of HuR are required.

Reviewer 2 Comments for the Author: [Major points]
1) The HuR-deficient neurons are accumulated in the IZ and VZ/SVZ. However, HuR expression is hardly observed in the IZ and VZ/SVZ at E15.5 (Fig. 1D). The authors should explain the inconsistent data. In addition, both multipolar and bipolar neurons are located at the IZ. It is needed to clarify which type of the migrating neurons expresses HuR. Response: We thank the reviewer for pointing out this issue. In our previous manuscript, we used an anti-HuR antibody from Abcam (ab136542) to visualize the expression of HuR in brain sections. We noticed that several previous studies that examined HuR expression in developing brains all used antibody from Santa Cruz Biotechnology (Kraushar et al., PNAS, 2014, Popovitchenko et al., Scientific Reports, 2016; Wang et al., Cell Reports, 2019). During the revision of this manuscript, we reexamined the HuR expression in developing and developed mice brains using the anti-HuR antibody from Santa Cruz (sc5261).
The new results showed, HuR had ubiquitous expression in the E16.5 developing neocortex, which was consistent with previous reports using the same antibody (Kraushar et al., PNAS, 2014, Popovitchenko et al., Scientific Reports, 2016; Wang et al., Cell Reports, 2019). To answer if HuR expressed in migrating neurons, we co-stained HuR with DCX, which is a marker of immature neurons. Result showed HuR co-localized with DCX at both IZ and CP in the developing brain. Please see lines 95-97 and Fig.1C.
Since HuR highly co-localized with DCX in the low and up IZ of E16.5 brain sections, we suspected that HuR expressed in both multipolar and bipolar neurons in the IZ. In order to further answer this question, we stained HuR in shNC electroporated brain sections. HuR expression was found in both multipolar neurons and bipolar neurons in the IZ (see Figure below). However, the result in this manuscript did not indicate HuR may have different roles in these two types of neurons. And the co-localization of HuR and DCX already indicated that HuR should express in both types of neurons. Therefore, we did not put this part of result in the revised manuscript. We hope the reviewer could agree with us on this point.

© 2020. Published by The Company of Biologists under the terms of the Creative Commons Attribution License (https://creativecommons.org/licenses/by/4.0/).

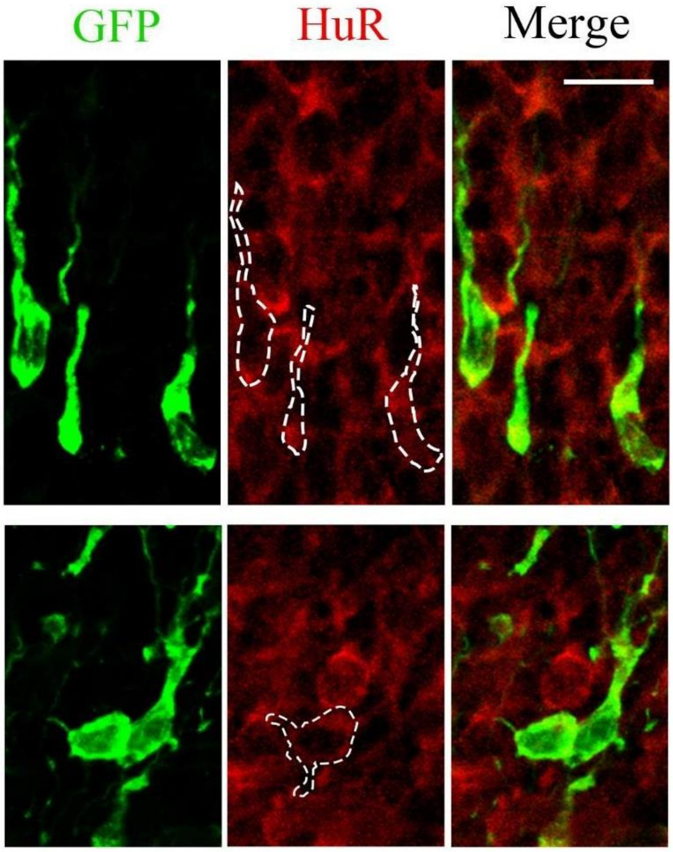

Figure. HuR expression in multipolar and bipolar neurons.
The shNC plasmid was electroporated into E15.5 WT mice cortex, and brain slices were prepared at E18.5. Brain slices were stained with anti-GFP and anti-HuR antibodies. Expression of HuR was observed in both multipolar and bipolar neurons.

2) The authors also mentioned that HuR is expressed in the CDP-positive neurons. However, the expression of HuR is weak in the CDP-positive upper layers at E18.5 (Fig. 1D). These images may indicate that HuR is expressed in the mature deep layer neurons (but less expressed in the migrating and upper layer neurons).
Response：Thank you for the reviewer to point out this issue. We noticed that co-staining HuR with CDP (or CTIP2) in E18.5 (E15.5) brain slice was somehow misleading in this manuscript, because we were trying to study the function of HuR in the migrating immature neurons. However, CDP and CTIP2 are both markers for mature neurons.
During the revision of this manuscript, we have replaced this staining image. The major purpose of this manuscript was to study the function of HuR in migrating neurons. Therefore, we co-stained HuR with DCX to see if HuR expressed in the migrating neurons. Please see revised Fig.1C
We did co-stained HuR with CDP (CTIP2) in the postnatal (P14) mice brains. In WT brains, HuR expressed in both CDP and CTIP2 positive cells in the cortex. Please see Fig.S2C.

3) As I mentioned above, profilin expression barely rescued the migration defects of the HuR-deficient neurons. The migration distance of the HuR-deficient neurons is 30-40 um (Fig. 3E), but the profilin1-electroporated neurons is around 20 um (Fig. 5G), suggesting that profilin1 could not rescue the migration defects. The authors should examine whether other downstream candidates, such as Arp2/3, cofilin and Rnd3 (from Table S1), rescue the phenotypes of the HuR-deficient neurons or not.
Response: The Reviewer compared the migration distance in Fig. 3E and Fig. 5G. However, these

© 2020. Published by The Company of Biologists under the terms of the Creative Commons Attribution License (https://creativecommons.org/licenses/by/4.0/).

two results were calculated from neurons that localized at different regions (Fig. 3E for migrating neurons in the CP and Fig. 5G for migrating neurons in the IZ). In our manuscript, Fig. 3B showed the migration distance of WT neurons in the IZ, which was 21.11±9.20 μm. The migration distance of Pfn1 rescued neurons in the IZ was 19.56± 10.93 μm. Therefore, we concluded that Pfn1 dramatically improved the cell motility of HuR-deficient neurons.
However, considering the broad effect of HuR, we fully agree that the migration defects in HuR deficient neurons may be resulted from multiple downstream mRNAs. To further study which downstream effector may also contribute to HuR regulated neuronal migration, we applied qPCR analysis to the HuR KO cortical samples. Among the top down-regulated genes in the PCR array, expression levels of *pfn1* and *arf6* were significantly reduced, whereas the expression of *actinin1* and *fap* were not significantly altered. Thus, we chose Arf6 for a second rescue experiment. Arf6 has been proved to affect the migration speed in the IZ (Hara et al., 2016). However, we found co-expression of Arf6 did not improve the migration defects in HuR KO neurons. These results had been added to the revised manuscript. Please see lines242-246 and Fig. S6E-G.

4) The HuR-deficient bipolar neurons seem to have an abnormally branched leading process. A recent report indicates that knockdown of Cav1, a HuR target gene candidate (Table S1), results in branched leading processes (iScience, 2018, 7:53-67). Because profilin expression may not rescue the branching phenotype, it would be better to analyze whether Cav1 is another functional target of HuR in the regulation of proper neuronal morphologies.
Response: We performed the rescue experiment as the reviewer suggested. The Cav1 expression vector was requested from Dr. Takeshi Kawauchi (Keio University School of Medicine). However, we found Cav1 overexpression did not rescue the migration defects in HuR KO neurons (see figure below). In addition, we also found the expression level of Cav1 did not significantly alter in HuR cKO cortex (see figure below). Therefore, the aforementioned results were not included in the revised manuscript. However, we did discuss if Cav1 may related to HuR mediated neuronal migration. Please see lines 312- 313.

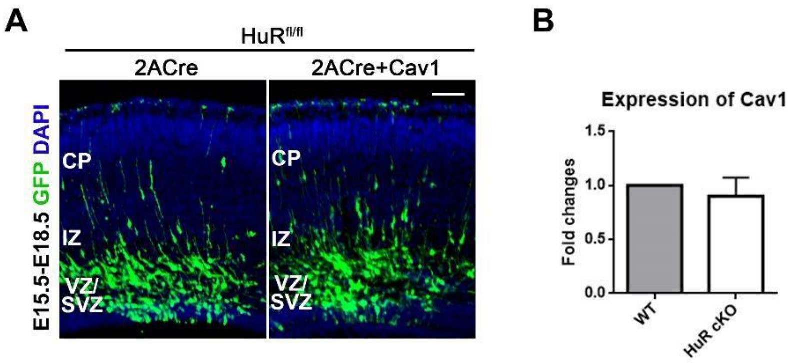

Figure. Cav1 overexpression did not rescue migration defects in HuR KO neurons
(A) E15.5 $HuR^{fl/fl}$ embryonic brains were electroporated with the indicated plasmids, and cortical slices were stained with an anti-GFP antibody and DAPI at E18.5. The mole ratio of 2A-Cre vector to Pfn1 expression vector is 1:1. Scale bar=100 μm.(B) qPCR analysis of Cav1 in P0 $HuR^{fl/fl}$; Nestin-Cre and $HuR^{fl/fl}$ mice cortical lysates. The expression level of Cav1 is not significantly reduced.

5) HuR deletion reduces the ratio of F-actin to total actin. The authors should examine whether profilin1 expression rescues this phenotype.
Response: We thank the reviewer for the constructive advice. We tried to rescue the actin polymerization in HuR deficient cells by co-expression with Pfn1. We found the ratio of F- actin fraction was decreased in HuR knockdown NLT cells. And the ratio of F-actin fraction was significantly elevated when Pfn1 was co-expressed with shHuR plasmid. Therefore, we conclude that the actin polymerization in HuR deficient cells could be rescued by Pfn1 overexpression. Please find these new results in lines 229-232 and Fig. S6A-B.

© 2020. Published by The Company of Biologists under the terms of the Creative Commons Attribution License (https://creativecommons.org/licenses/by/4.0/).

6) In Fig. 2H, the MAP2-positive cells were counted. Because MAP2 is a marker for mature neurons that finish the neuronal migration, the expression of MAP2 in the migrating neurons should be very low. It is needed to provide the high magnification images and the single color (red and green) images. Are the expression levels of MAP2 in the migrating neurons comparable with that of the surrounding mature neurons?
Response: As the reviewer mentioned, MAP2 was a marker of mature neuron. But we did not intend to see the expression level of MAP2 in GFP+ cells. Because the zones of VZ/SVZ, IZ and CP were identified by DAPI staining, we intended to use MAP2 as a marker to visualize the CP area. We hope the reviewer could understand and agree with us on this point.

[Minor points]
7) The authors mentioned that HuR deficiency disturbed neuronal polarity (Fig. 3G). Do they analyze the position of centrosome or golgi? If not, the authors should weaken the statements.
Response: In Fig.3G, we intended to show HuR deficient neurons tend to loss leading process. We agree that this phenotype may not closely relate to the polarity of migrating neurons. Therefore, the statement of this result has amended to "The HuR-deleted neurons were inclined to the loss and regeneration of the leading process". Please see lines 172- 173.

8) Profilin1 is known to control actin dynamics in cooperation with other actin-binding proteins, such as cofilin and Arp2/3. Table S1 indicates that HuR regulates the mRNA expression of several other actin-binding proteins (e.g. Arp2/3, cofilin, actinin1 and myosin-10) and actin-regulatory proteins (e.g. Rnd3, Rac1 and Cdc42). Among them, Arp2/3 (Development, 2016, 143(15):2741-2752), cofilin (Nat Cell Biol, 2006, 8(1): 17-26), myosin-10 (Cerebral Cortex, 2014, 24(5):1259-1268), Rnd3 (Neuron, 2011, 69(6):1069- 1084), Rac1 (EMBO J, 2003, 22(16): 4190-4201. Cell Rep, 2012, 2(3):640-651) and Cdc42 (J Biol Chem, 2005, 280(6):5082-5088. Nat Neurosci, 2006, 9(1):50-57) have been shown to regulate neuronal migration. It would be better to discuss the relationship between profilin1 and these molecules in neuronal migration.
Response: We thank the reviewer for this nice suggestion. We have carefully read these articles and compared the phenotypes that described in these papers with our data. The following content was added to the Discussion of our manuscript" In addition to Pfn1 and Arf6, many other genes that appeared in the PCR array regulate neuronal migration. For example, Rac1 and Cdc42 are small GTPases that are required for precise control during neuronal migration because constitutively active and dominant-negative forms of Rac1 and Cdc42 significantly inhibited radial migration (Konno et al., 2005). The functions of these two molecules were primarily related to malformation of the leading process (Kawauchi et al., 2003; Kholmanskikh et al., 2005; Yang et al., 2012). Rnd3 is an atypical Rho GTPase that antagonizes RhoA activity during neuronal migration. Rnd3-silenced neurons exhibit enlarged leading processes and an excess of thin processes that extend from the cell body and the leading process (Pacary et al., 2011). These GTPases regulate the leading process of migrating neurons, but these phenotypic defects were not present in HuR-deficient neurons, which indicates that the functions of these GTPases did not overlap with HuR or Pfn1. Similar to Pfn1, several actin-binding proteins also play roles in neuronal migration. Myo10 is an nontraditional myosin family member that is involved in establishing proper migration orientation and the multipolar-to-bipolar morphology transition (Ju et al., 2014). The Arp2/3 complex is an actin nucleator that produces branched actin networks, and it is essential for the maintenance of radial glial cells (RGC) polarity and organization (Wang et al., 2016). Caveolin-1 regulates clathrin-independent endocytosis and elongation of the leading process (Shikanai et al. 2018). Cofilin disassembles actin filaments, and it is negatively regulated by the CDK5-p27 pathway. An appropriate balance of cofilin phosphorylation is required for proper cortical neuronal migration (Kawauchi et al., 2006). However, these phenotypic defects were not similar to our observations in HuR-deficient neurons and brains. Notably, the expression of most of the aforementioned genes was not significantly changed in our PCR array. Therefore, HuR may not regulate the mRNA stability of these genes, and these genes should not play significant roles in HuR-mediated neuronal migration." Please see lines 296-320.

Reviewer 3 Advance Summary and Potential Significance to Field:
The manuscript "Human Antigen R regulated mRNA metabolism is essential for neuronal migration during neocortex development" describes the function of the HuR proteins in neuronal migration during corticogenesis. The authors analyzed the function of HuR proteins in cortical development

© 2020. Published by The Company of Biologists under the terms of the Creative Commons Attribution License (https://creativecommons.org/licenses/by/4.0/).

and radial neuronal migration. They found that HuR proteins are expressed in the developing cortical plate, especially in post-mitotic neurons. Genetical deletion of HuR gene in post-mitotic neurons using NEX-Cre affected the thickness of CDP-positive late-born neuron layer. They demonstrated that HuR proteins are important for normal radial migration of late-born neurons by BrdU injection and IUE experiments. Then they performed time-lapse imaging of HuR depleted neurons and found that migration distances were decreased compared to the control neurons. Using cell motility PCR array, they have screened for binding partners for HuR and found that profilin 1 is a strong candidate. So, they examined if HuR can stabilize mRNA of Profilin 1 and found that HuR is critical for mRNA stability of profilin 1. Finally, they indicated that Profilin 1 can rescue the migration defect phenotypes of HuR depleted neurons in terms of their migration distance and cell motility from which they lead to the conclusion that HuR is required to promote the cell motility via stabilization of profilin 1 mRNA during radial migration process.

This study provides interesting insights into HuR-Pfn1 dependent regulatory mechanism of radial neuronal migration during corticogenesis. However, several points need to be addressed prior publication in order to support the authors' conclusions:

Reviewer 3 Comments for the Author:

1. Fig 1 A-D

The authors claim that HuR proteins are localized in projection neurons in cortical plate because projection neurons were the major cell type in the cortical plate (line 80). However, GABA-positive inhibitory neurons should also be in the cortex although the cell number is low in this embryonic stage. They should check if GABAergic neurons have HuR proteins in the different embryonic or postnatal stages to reach their conclusion.

Response: We have tried to co-localize HuR with GABA in E16.5 and P7 cortices. However, there were very scarce GABA+ cells in the E16.5 developing cortex, whereas HuR expression was quite ubiquitous (Data not shown). Therefore we could not reach a confident conclusion if HuR co-localize with GABA in the developing cortex. In contrast, we detected strong GABA staining signal in P14 cortex and almost all GABA+ cells are also HuR+ (Figure below).

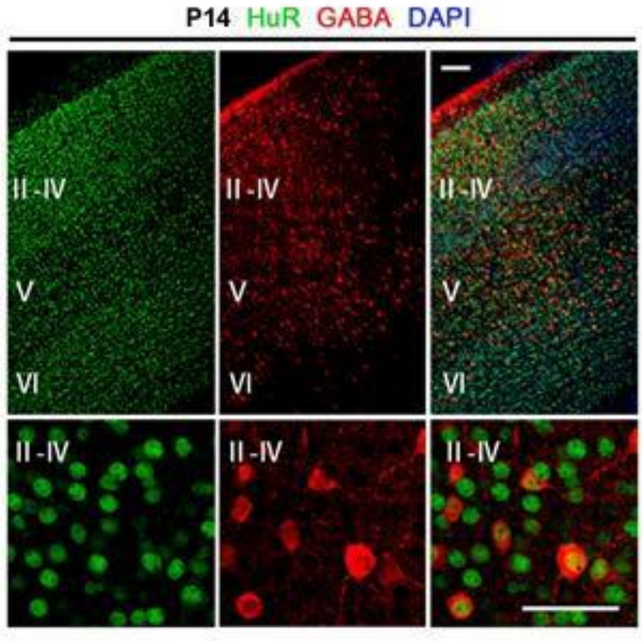

Figure. Expression of HuR and GABA in P14 WT cortex

In the current manuscript, we focused our study on neuronal migration of projection neurons. We used NEX-Cre to specifically knockout HuR in post-mitotic projection neurons. We showed HuR expression was dramatically decreased in P14 HuR$^{fl/fl}$; NEX-Cre cortex, and almost no HuR expressed in CDP+ and CTIP2+ cells (Fig. S2C). Although there were still HuR+ cells in P14 HuR$^{fl/fl}$; NEX-Cre cortex, signal may be from glia cells and interneurons. However, as we mentioned earlier, we did

© 2020. Published by The Company of Biologists under the terms of the Creative Commons Attribution License (https://creativecommons.org/licenses/by/4.0/).

not intend to study the expression and function of HuR in interneurons and glia cells. Therefore, we did not add the GABA staining result to the manuscript.
During the revision of this manuscript, a new paper (Wang et al. 2019 Cell Reports) showed HuR deletion did not impair proliferation and differentiation of NPCs during embryonic development. And we further found HuR co-localizes with the immature neuron marker DCX. Therefore, we had further evidence to show HuR has a major role in neuronal migration of projection neurons. The statement “projection neurons were the major cell type in the cortical plate” was deleted from the manuscript. We hope the reviewer could agree with us on this point.

2. Fig S2
They claim that HuR deletion did not affect neural progenitor cell proliferation and differentiation by immunostaining of PAX6, Tbr2 and Ki67. These pictures in Fig S2-A, there are not so many GFP positive neurons in the ventricular zone compared to Fig 2E.

They should show the whole sections of Fig S2A in supplemental data.
Response: In the revision of this manuscript, we applied new experiments to study if HuR deletion has any effect on the fate of neural progenitor cells. We utilized $HuR^{fl/fl}$; Nestin- Cre mice, which deleted HuR in all types of neural progenitor cells. BrdU was injected at E14.5 and brain slices were prepared in 2 hours and 48 hours, respectively. Results showed the ratios of PAX6+ or Tbr2+ cell among total BrdU+ cell were similar between $HuR^{fl/fl}$; Nestin-Cre and $HuR^{fl/fl}$ mice in 2 hours post BrdU injection. And the ratio of Tbr1+ cell among total BrdU+ cell was also similar between $HuR^{fl/fl}$; Nestin-Cre and $HuR^{fl/fl}$ mice in 48 hours post BrdU injection.

During the revision of this manuscript, a paper was published from our collaborator (Dr. Weixiang Guo, who is also a correspondence author of this manuscript). In their work, they studied the functions of HuR in neurogenesis during embryonic and adult stages. Their results also showed that HuR conditional deletion by EMX1-Cre did not affect embryonic neurogenesis (Wang et al., 2019, Cell Reports, DOI:10.1016/j.celrep.2019.10.127). Altogether, our new results and data from Dr. Guo’s work revealed that HuR did not regulate neurogenesis during embryonic development.

As the reviewer suggested, the whole cortex images were used in the revised manuscript. For detail of these results please see revised Fig. S1.

3. Fig 3
There is only one trace of migrating cell in each live imaging. They should show more several traces for each condition as supplemental data. 8hrs time-lapse imaging is not sufficient for observing the migration status. Can they demonstrate any longer imaging data? Moreover, they didn’t describe how to calculate the migration distances from the movie data in the method section. They should specify the method including the name of the analyzing software. If they didn’t use any software, they should show the several examples of hand tracing lines they measured. About the graph G, they also should mention how to measure the % of “loosing polarity”. About the numbers of measured samples, the definition is not clear. They should state the detailed brain number or slice number and how many times of the same independent experiments.
Response:
As the reviewer suggested, we have added more tracing of the migrating neurons in the supplemental figures. Please see Fig. S4A and F.
Regarding the duration of time-lapse imaging, we have done longer imaging in our previous work (up to 15 h, Ju et al., 2014, Cerebral Cortex, 24:1259–1268). In our experience, an 8-h time-lapse imaging is already sufficient to calculate the migration speed of a migrating neuron. Comparing with our own data, the migration speed form an 8-h time- lapse imaging (data in this manuscript and data in Zhang et al., 2018, Cell Reports, 22(13), 3598-3611) is consistent with that from a 15-h time-lapse imaging (Ju et al., 2014, Cerebral Cortex, 24:1259–1268). 8 h time-lapse imaging is also common in such study, such as that reported in Bamat et al., Neuron, 2017, 93, 99–114. We hope the reviewer could agree with us on this point.
We are sorry that we did not explain how we calculate the migration distance in detail. We have added the following information in the Method part. See lines 553-556. Time-lapse images were acquired every 20 min. Among all images, a total of 9 images were used for calculating migration distance (0 min to 480 min, 60 min intervals). For a given neuron, the center point of this neuron

© 2020. Published by The Company of Biologists under the terms of the Creative Commons Attribution License (https://creativecommons.org/licenses/by/4.0/).

was located in each image. Then ImageJ was used to generate a tracing line that connects these center points in sequence, and the length of this tracing line was calculated by ImageJ. Please see the following figure for an example of a migration tracing line.

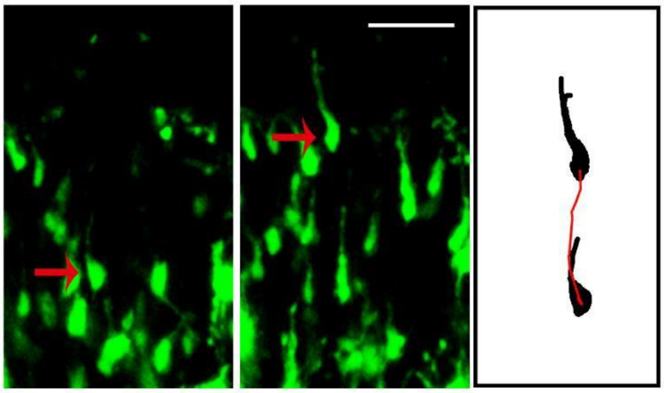

Figure Migration tracing line of a neuron in 8 h
The 0 min and 480 min images of the same cortical section were shown. The cell tracing and migration line tracing (red line) were shown in the right panel.

For Fig. 3G, we measured the percentage of GFP+ cells that lost leading process or generated extra process during the recording period. This method was adopted from Bamat et al., Neuron, 2017. In addition, as another reviewer suggested that we did not examine the cell polarity by studying Golgi or centrosome. We have amended the statement to "% of GFP$^+$ neurons lost leading process". For this experiment, we performed 4 independent experiments and at least 40 GFP+ cells were counted in each cortical slice. This information was added to the Fig. 3 legend.

4. The reason they chose pfn1 as a candidate for binding partner of HuR was not clear. According to their gene expression data of cell motility PCR array, I found that Actn1, Arf6 and Arhgef 7 are highly downregulated or upregulated compared to pfn1. All of them are related to actin dynamics and cell motility, suggesting the possibility that they also contributed to the phenotype shown in HuR knockout neurons. How they chose only pfn1 to further analysis is not clear to me. Please mention about this point in the manuscript.
Response:
In our manuscript, we first chose Pfn1 for rescue experiment based on two reasons. First, Pfn directly regulates actin dynamics to mediate cell motility. Second, phenotype of pfn1 KO mice was similar to that of HuR KO mice.
However, as this and other reviewers pointed out the expression levels of many other genes were also altered. To further study if other downstream effectors may also contribute to HuR regulated neuronal migration, we applied qPCR analysis to the HuR KO cortical samples. Among the top down-regulated genes in the PCR array, expression levels of *pfn1* and *arf6* were significantly reduced, whereas the expression of *actinin1* and *fap* were not significantly altered. Thus, we chose Arf6 for a second rescue experiment. Arf6 has been proved to affect the migration speed in the IZ (Hara et al., 2016). However, we found co- expression of Arf6 did not improve the migration defects in HuR KO neurons. These results had been added to the revised manuscript. Please see lines 242-246 and Fig. S6E-G.

5. Fig4
Pfn1 mRNA stability is decreased when HuR proteins are depleted (F). Is there any mRNA of which the stability is not affected by HuR depletion? They should indicate this example as a negative control.
Response: During the revision of this paper, we performed qPCR analysis using P0 cortical RNA samples. We firstly analyzed the expression levels of several house-keeping genes, included 18s RNA, GAPDH and Tbp1. We found the expression levels of these genes were maintained similar between HuR$^{fl/fl}$; Nestin-Cre and HuR$^{fl/fl}$ cortices, indicating HuR may not regulate the expression of these

© 2020. Published by The Company of Biologists under the terms of the Creative Commons Attribution License (https://creativecommons.org/licenses/by/4.0/).

genes. Consistent with our finding, previous result from other publication has shown the expression of GAPDH was not regulated by HuR (Yoon et al., Nucleic Acids Research, 2014, 42(2), 1196-1208). Therefore, we used GAPDH as the internal control in our qPCR analysis.

6. Fig 5
Again, they should show more examples of cell traces for migrating behavior as a supplemental data.
Response: We have added more examples of cell traces in the revised manuscript. Please see revised Fig. S4A and F.

7. They claim that the MP-BP transition is not impaired but the motilities of bipolar shape is decreased. However, it's hard to convince people from the present data. Please show other pictures so that they can see the author's claim.
Response: In our experiment, the Cre-GFP vector restricted GFP expression in nuclei, which cannot be used for morphology analysis. And the time-lapse images were also not ideal for such analysis. To cope with this issue, we added an additional data set, in which HuR knockdown was performed in migrating neurons. The GFP signal in the pGeneClip™ hMGFP backbone showed cytoplasmic distribution, which is suitable for morphology analysis of the migrating neurons. Results from HuR knockdown assay revealed that the multipolar-to-bipolar transition in the IZ was not impaired. But the neuronal migration defects maintained. We think this new data could support our conclusion that the cell motility defect rather than the multipolar-to-bipolar transition defect was the major causal factor to delay neuronal migration. Please find these new data in lines 161-165 and Fig. S4B-E.

Minor points
1. In the introduction part, you cited Ayala et al., 2007 to explain the radial migration process. However, there are more recent review such as "Ohtaka-Maruyama and Okado, Frontiers in Neuroscience, 2015 Dec 17;9:447.2018" is also recommended.
Response: We thank the reviewer for pointing out this useful paper. It has been cited in the revised manuscript. Please see line 47.
2. The reason for that the migration of later-born neurons are more affected than deep layer neurons although HuR is expressed in the both cell population should be discussed in the discussion part.
Response: We have elaborated the discussion to address why HuR has more prominent impact on late-born neurons. The following content was added: We found that HuR deletion impaired proper cortex formation, which was shown by the reduced thickness of layers II-IV. In contrast, the deeper layers of the cortex were maintained. Consistent with these data, the BrdU birth-dating assays showed that the migration of late-born neurons (E15.5), but not early-born neurons (E12.5), impaired significantly. Two reasons may account for the differential roles HuR plays in early-born and late-born neurons. The protein levels of HuR were most prominent in E15.5 and E17.5 brains, which corresponds to the development of late-born neurons. Therefore, HuR should have a more sophisticated impact in these neurons. Notably, we found that the cortical lamination defects in $HuR^{fl/fl}$; Nex-Cre brains primarily resulted from the impaired cell motility of HuR KO neurons. During brain development, late-born neurons travel a much longer distance to arrive at their proper positions in the cortex. Therefore, the requirement for HuR-mediated cell motility was more imperative in these cells. Please see lines 265-276.
3. About the rescue experiment of HuR deleted neurons by Pfn1, the authors should try several different concentrations of Pfn1 expression plasmids because the overexpression of Pfn1 impairs the radial migration (Fig.S4B). In this regards, they should specify the concentration of rescue plasmid concentration in the method part instead of describing "in designed dosage" (line315).
Response: In our initial rescued experiments we tried two different concentrations of Pfn1. The mole ratio of Cre vector to Pfn1 vector was 2:1 and 1:1, respectively. Though co- expression of Pfn1 at both concentrations improved neuronal migration of HuR KO neurons (figure below), the higher concentration Pfn1showed better effect, which almost fully rescued the GFP+ cell distribution defects. Since we have also found overexpression of Pfn1 alone impaired neuronal migration, we did not try higher concentrations of Pfn1. The result showed in the manuscript was the concentration that had the best effect in our test. We have added the concentration information into the revised figure legend and added the strategy for screening the best rescue concentration in the method part. See lines 417-420. This strategy also applied to the rescue experiments that were performed during

© 2020. Published by The Company of Biologists under the terms of the Creative Commons Attribution License (https://creativecommons.org/licenses/by/4.0/).

this revision.

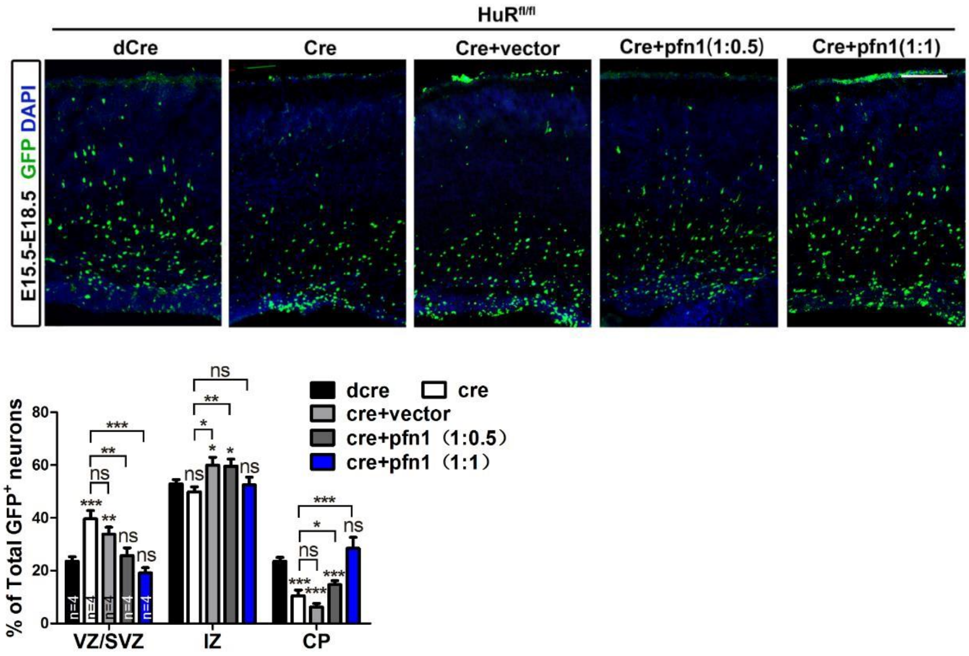

<u>Second decision letter</u>

MS ID#: DEVELOP/2019/183509

MS TITLE: Human Antigen R-regulated mRNA metabolism promotes the cell motility of migrating neurons

AUTHORS: Xiaoxiao He, Yifei Zhao, Zifei Song, Yanning Zhang, Ye Guo, Huali Yu, Zixuan He, Wencheng Xiong, Weixiang Guo, and Xiaojuan Zhu

I have now received the reports of the three referees who reviewed the earlier version of your manuscript and I have reached a decision. The referees' comments are appended below, or you can access them online: please go to BenchPress and click on the 'Manuscripts with Decisions' queue in the Author Area.

The reviewers' evaluation is positive and we would like to publish a revised manuscript in Development, provided that you satisfactorily address the remaining comment of referee 2. Please attend to all these comments in your revised manuscript and detail them in your point-by-point response. If you do not agree with any of their criticisms or suggestions explain clearly why this is so.

<u>Reviewer 1</u>

Advance summary and potential significance to field

The authors have addressed most of the previous concerns in a satisfactory manner.

Comments for the author

The images relating to multipolar neurons do not allow to easily resolve the exact neuronal morphology.

© 2020. Published by The Company of Biologists under the terms of the Creative Commons Attribution License (https://creativecommons.org/licenses/by/4.0/).

Reviewer 2

Advance summary and potential significance to field

In the revised version of the manuscript, the authors adequately address most of my previous concerns. There are, however, two minor issues that require further attention.

Comments for the author

1) According to the authors' response to my previous comment-6, the authors counted the GFP-positive cell number in the MAP2-positive CP area, but not the GFP- and MAP2-double positive cell number. However, the authors still mention "significantly fewer cells presented in the CP of HuR KO brains, which were revealed by co-staining GFP with MAP2 (Fig. 2G and H)" in the revised manuscript and "GFP+ MAP2+/total GFP+ (%)" in Fig. 2H. Again, migrating neurons are basically negative for MAP2ab, and therefore the authors should rewrite these statements.

2) I found a typo in the last sentence of the Abstract; "brian development" should be changed into "brain development".

Reviewer 3

Advance summary and potential significance to field

They analyzed the function of HuR proteins in neuronal migration. They found that HuR proteins are expressed in post-mitotic neurons. Then they found HuR can stabilize mRNA of Profilin1. Moreover, Profilin 1 can rescue the migration defect phenotypes of HuR depleted neurons.

This study shows HuR-Pfn1 dependent regulatory mechanism of radial neuronal migration. This new finding provides interesting insights into the mechanism of radial neuronal migration via controlling mRNA stability of downstream partner.

Comments for the author

The authors tried to clarify the points indicated by reviewers. They added new experiments to convince reviewers. They answered my concerns and I have no further comments.

Second revision

Author response to reviewers' comments

Reviewer 1 Advance summary and potential significance to field
The authors have addressed most of the previous concerns in a satisfactory manner.

Reviewer 1 Comments for the author
The images relating to multipolar neurons do not allow to easily resolve the exact neuronal morphology.

Response: To deal with this issue, we tried to further magnify the confocal images. We hope this change could help to show the neuronal morphology clearly. Please see revised Fig. S4B.

Reviewer 2 Advance summary and potential significance to field
In the revised version of the manuscript, the authors adequately address most of my previous concerns. There are, however, two minor issues that require further attention.

© 2020. Published by The Company of Biologists under the terms of the Creative Commons Attribution License (https://creativecommons.org/licenses/by/4.0/).

Reviewer 2 Comments for the author
1) According to the authors' response to my previous comment-6, the authors counted the GFP-positive cell number in the MAP2-positive CP area, but not the GFP- and MAP2-double positive cell number. However, the authors still mention "significantly fewer cells presented in the CP of HuR KO brains, which were revealed by co-staining GFP with MAP2 (Fig. 2G and H)" in the revised manuscript and "GFP+ MAP2+/total GFP+ (%)" in Fig. 2H. Again, migrating neurons are basically negative for MAP2ab, and therefore the authors should rewrite these statements.

Response: We thank the reviewer to point out this issue again. As we have explained in the previous response, we did not intend to look at the GFP+ MAP2+ double positive staining rate. Instead, we wanted to show how many GFP+ cells entered the CP, which is represented by MAP2 staining.
We have re-writed the text and the figure regarding to this issue. The text has been amended as "Correspondingly, significantly fewer GFP+ cells presented in the CP (represented by MAP2 staining) of HuR KO brains (Fig. 2G and H)." See lines 137-138. The Y axis of Fig. 2H was amended as "GFP+ in MAP2+ zone / total GFP+ (%)".

2) I found a typo in the last sentence of the Abstract; "brian development" should be changed into "brain development".

Response: We are sorry for this mistake. During the revision, we found this sentence is redundant for the Abstract. Therefore, it has been deleted from the manuscript. But we did find two similar typo issues, we have corrected the mistakes.

Reviewer 3 Advance summary and potential significance to field
They analyzed the function of HuR proteins in neuronal migration. They found that HuR proteins are expressed in post-mitotic neurons. Then they found HuR can stabilize mRNA of Profilin1. Moreover, Profilin 1 can rescue the migration defect phenotypes of HuR depleted neurons.

This study shows HuR-Pfn1 dependent regulatory mechanism of radial neuronal migration. This new finding provides interesting insights into the mechanism of radial neuronal migration via controlling mRNA stability of downstream partner.

Reviewer 3 Comments for the author

The authors tried to clarify the points indicated by reviewers. They added new experiments to convince reviewers. They answered my concerns and I have no further comments.

Response: We thank the constructive comments from the reviewer.

Third decision letter

MS ID#: DEVELOP/2019/183509

MS TITLE: Human Antigen R-regulated mRNA metabolism promotes the cell motility of migrating neurons

AUTHORS: Xiaoxiao He, Yifei Zhao, Zifei Song, Yanning Zhang, Ye Guo, Huali Yu, Zixuan He, Wencheng Xiong, Weixiang Guo, and Xiaojuan Zhu
ARTICLE TYPE: Research Article

I am delighted to tell you that your manuscript has been accepted for publication in Development, pending our standard ethics checks.

© 2020. Published by The Company of Biologists under the terms of the Creative Commons Attribution License (https://creativecommons.org/licenses/by/4.0/).
